# Blockage of VEGF function by bevacizumab alleviates early-stage cerebrovascular dysfunction and improves cognitive function in a mouse model of Alzheimer’s disease

**DOI:** 10.1186/s40035-023-00388-4

**Published:** 2024-01-03

**Authors:** Min Zhang, Zhan Zhang, Honghong Li, Yuting Xia, Mengdan Xing, Chuan Xiao, Wenbao Cai, Lulu Bu, Yi Li, Tae-Eun Park, Yamei Tang, Xiaojing Ye, Wei-Jye Lin

**Affiliations:** 1 Brain Research Center, Sun Yat-sen Memorial Hospital, Sun Yat-sen University, Guangzhou, 510120 China; 2 Faculty of Forensic Medicine, Zhongshan School of Medicine, Sun Yat-sen University, Guangzhou, 510120 China; 3 Guangdong Province Translational Forensic Medicine Engineering Technology Research Center, Sun Yat-sen University, Guangzhou, 510120 China; 4 Guangdong Provincial Key Laboratory of Brain Function and Disease, Zhongshan School of Medicine, Sun Yat-sen University, Guangzhou, 510120 China; 5 Department of Neurology, Sun Yat-sen Memorial Hospital, Sun Yat-sen University, Guangzhou, 510120 China; 6 Guangdong Provincial Key Laboratory of Malignant Tumor Epigenetics and Gene Regulation, Guangdong-Hong Kong Joint Laboratory for RNA Medicine, Medical Research Center, Sun Yat-sen Memorial Hospital, Sun Yat-sen University, Guangzhou, 510120 China; 7 Nanhai Translational Innovation Center of Precision Immunology, Sun Yat-sen Memorial Hospital, Foshan, 528200 China; 8 Department of Biomedical Engineering, College of Information and Biotechnology, Ulsan National Institute of Science and Technology (UNIST), Ulsan, 44919 Republic of Korea

**Keywords:** Alzheimer’s disease, Bevacizumab, Vascular endothelial growth factor, Cerebrovascular function

## Abstract

**Background:**

Alzheimer's disease (AD) is a neurodegenerative disorder and the predominant type of dementia worldwide. It is characterized by the progressive and irreversible decline of cognitive functions. In addition to the pathological beta-amyloid (Aβ) deposition, glial activation, and neuronal injury in the postmortem brains of AD patients, increasing evidence suggests that the often overlooked vascular dysfunction is an important early event in AD pathophysiology. Vascular endothelial growth factor (VEGF) plays a critical role in regulating physiological functions and pathological changes in blood vessels, but whether VEGF is involved in the early stage of vascular pathology in AD remains unclear.

**Methods:**

We used an antiangiogenic agent for clinical cancer treatment, the humanized monoclonal anti-VEGF antibody bevacizumab, to block VEGF binding to its receptors in the 5×FAD mouse model at an early age. After treatment, memory performance was evaluated by a novel object recognition test, and cerebral vascular permeability and perfusion were examined by an Evans blue assay and blood flow scanning imaging analysis. Immunofluorescence staining was used to measure glial activation and Aβ deposits. VEGF and its receptors were analyzed by enzyme-linked immunosorbent assay and immunoblotting. RNA sequencing was performed to elucidate bevacizumab-associated transcriptional signatures in the hippocampus of 5×FAD mice.

**Results:**

Bevacizumab treatment administered from 4 months of age dramatically improved cerebrovascular functions, reduced glial activation, and restored long-term memory in both sexes of 5×FAD mice. Notably, a sex-specific change in different VEGF receptors was identified in the cortex and hippocampus of 5×FAD mice. Soluble VEGFR1 was decreased in female mice, while full-length VEGFR2 was increased in male mice. Bevacizumab treatment reversed the altered expression of receptors to be comparable to the level in the wild-type mice. Gene Set Enrichment Analysis of transcriptomic changes revealed that bevacizumab effectively reversed the changes in the gene sets associated with blood–brain barrier integrity and vascular smooth muscle contraction in 5×FAD mice.

**Conclusions:**

Our study demonstrated the mechanistic roles of VEGF at the early stage of amyloidopathy and the protective effects of bevacizumab on cerebrovascular function and memory performance in 5×FAD mice. These findings also suggest the therapeutic potential of bevacizumab for the early intervention of AD.

**Supplementary Information:**

The online version contains supplementary material available at 10.1186/s40035-023-00388-4.

## Introduction

Alzheimer's disease (AD) is an irreversible neurodegenerative disease and a major cause of dementia that typically manifests in old or late middle age, with higher incidence in women than in men [1, 2]. The main symptoms of AD include progressive memory loss, language deficits and difficulty concentrating, which eventually impede one’s ability to perform everyday tasks [3, 4]. Pathological features of AD include deposition of amyloid beta (Aβ), formation of neuronal fibrillary tangles, activation of glial cells, and neuronal loss [5–8]. Although the underlying pathological mechanisms have been studied for decades, effective treatments for AD are still lacking.

Patients with diseases related to pathological changes in vascular function, including hypertension, type 2 diabetes, and atherosclerosis, are at a higher risk of developing AD and other types of cognitive disorders [9, 10]. Adequate blood supply is needed for the maintenance of brain function. Vascular dysfunction, which involves the loss of the structural or functional integrity of blood vessels, has been shown to play a critical role in the pathogenesis of AD [11, 12]. In AD patients and 16- to 18-month-old APP/PS1 mice, a 10%–30% reduction of cerebral blood flow (CBF) and increased blood–brain barrier (BBB) permeability have been reported, which are considered potential biomarkers for predicting the progression and severity of AD [13–16]. In addition to diseased brains, the normal aging population also show reduced resting CBF in the hippocampus, which is associated with worsened spatial memory performance [17–19]. The mechanistic link between CBF abnormalities and AD as well as its relationship with vascular endothelial growth factor (VEGF) function has been recently studied [11].

VEGF is involved in the regulation of a broad variety of physiological functions of blood vessels, including angiogenesis, vascularization, and permeability [20, 21]. Increased levels of VEGF have been detected in the plasma or brain parenchyma of AD patients and 10- to 14-month-old AD mice of both sexes, and have been linked to microvascular leakage and reduced blood flow possibly through binding and activation of VEGF receptor 2 (VEGFR2), which was also found to be increased in the cortex of AD mice [22–26]. Peripheral administration of anti-VEGF antibody in 10- to 14-month-old APP/PS1 mice caused both acute and chronic reduction of cortical capillary stalls and vascular permeability, and increase of blood flow [11]. Although the above study suggests that luminal VEGF may participate in the onset of microvasculature pathology in the middle-aged APP/PS1 mouse model, contradictory findings of neuroprotection by VEGF have also been reported [27, 28]. Thus, the impact of VEGF on the disease progression of AD remains uncertain. VEGF receptor 1 (VEGFR1) is considered a decoy receptor that competes with VEGFR2 for affinity binding to VEGF. It exists in membrane-bound full-length and short soluble forms [23, 29]. Notably, decreased levels of VEGFR1 were observed in the parietal cortex of AD patients and in the brains of 10- to 11-month-old APP/PS1 mice, suggesting an imbalance toward VEGFR2-mediated effects in the brain parenchyma of AD [11, 23]. Whether VEGF-mediated vascular pathology or neuroprotection exists in the early stage of AD remains elusive.

5×FAD mice are a commonly used amyloidopathy model of AD that carry five familial AD mutation sites in the human *APP* and *PSEN1* genes and rapidly develop Aβ plaques and cognitive deficits across multiple domains from 4–5 months of age [30–32]. Vatalanib, a small-molecule, broad-spectrum tyrosine kinase inhibitor (TKI) that exerts inhibitory effects on VEGFR1, VEGFR2, platelet-derived growth factor receptor α, and c-KIT, has been shown to significantly reduce Aβ plaque numbers and phosphorylated Tau proteins in the cortex of 3-month-old male 5×FAD mice after being administered daily for 14 days [33]. However, whether specifically targeting VEGF function at an early age will affect vascular pathology and, importantly, memory performance in 5×FAD mice, and whether sex-specific differences exist still await elucidation.

In the current study, we aimed to explore the functional roles of VEGF in 4- to 5-month-old 5×FAD mice by specifically antagonizing VEGF function with systematic administration of bevacizumab, a humanized monoclonal antibody against VEGF and the most clinically advanced antiangiogenic agent used as the first-line treatment for cancer and macular degeneration [34], which prevents VEGF binding to its receptors. The cerebrovascular functions, brain pathology, and long-term memory were examined in both sexes of mice. Protein levels of VEGF and its binding receptors were investigated, together with the elucidation of bevacizumab-associated transcriptomic alterations in the hippocampus of 5×FAD mice by RNA-sequencing analysis. Collectively, our findings suggest a mechanistic role of VEGF in the early stage of AD pathology and the therapeutic potential of bevacizumab for early intervention in AD.

## Materials and methods

### Animals

The 5×FAD mice (B6/SJL genetic background, JAX#034840) overexpressing both human APP harboring the Swedish (K670N and M671L), Florida (I716V) and London (V717I) FAD mutations, and the PS1 harboring the two FAD mutations (M146L and L286V) were obtained from the Jackson Laboratory (Bar Harbor, ME) and backcrossed to the C57BL/6 J genetic background [32]. A total of 176 mice were used in this study, including 98 female and 78 male mice. Drug administration began at 4 months of age for 5×FAD mice and their wild-type littermates. Experiments for testing cognitive performance, vascular function, and gene expression profiles were performed at the same time point in different cohorts of mice. The mice were housed in groups of 4–5 in an environmentally controlled animal facility on a 12 h light/dark cycle. Food and water were available ad libitum. All animal studies were approved by the Institutional Animal Care and Use Committee of the Sun Yat-sen University.

### Experimental procedures and drug treatment

Mice at 4 months of age received intraperitoneal injection of bevacizumab (10 mg/kg body weight) or 0.9% saline twice a week for one month [35, 36]. Behavioral tests, two-photon imaging, and cerebral vascular function analysis were performed at the end of the bevacizumab treatment. Before conducting behavioral tests, all the mice were handled for five days to reduce their anxiety level.

### Novel object recognition (NOR) task

The NOR task was modified based on previous studies [37, 38]. Briefly, the tests were carried out in a 40 × 40 × 40 cm^3^ arena placed in a quiet room with dim light. During the habituation session on the first day, the mice were allowed to explore the arena without objects for 10 min. On the second day, the mice explored the same arena again for 10 min, with two objects placed towards the two ends of a side wall. On the third day, the mice were tested for their long-term memory for the novel object. During the test session, the mice were allowed to explore the arena for 5 min, with one of the old objects replaced by a new object. Animal behaviors were videotaped, and the exploration time was scored by investigators blind to the experimental conditions. The traveling distance was analyzed by the TopScan software (CleverSys Inc., Reston, VA).

### Open field test

The test was modified based on a previous study [39]. The exploratory activity of each mouse in a 40 × 40 × 40 cm^3^ arena was indicated by the total distance and the time exploring the arena. The anxiety-like behaviors were assessed as the percentages of the distance and time spent in the 20 × 20 cm^2^ center zone to the total distance and time, respectively. Data were analyzed by the TopScan software (CleverSys Inc.).

### In vivo two-photon microscopy

A mouse was anesthetized with sodium pentobarbital (100 mg/kg body weight) and positioned on a brain stereotaxic frame (RWD Life Science, Shenzhen, China). Throughout the experiment, the mouse was kept on a temperature-controlled heating pad set at 37 ℃ to maintain their body temperature. The cranial window method was employed to measure the presence of stalled neutrophils in the cortical capillaries [40, 41]. Blood vessels were labelled by 70 kDa Texas Red dextran (5 mg/ml, Thermo Fisher, cat#: D1830, Waltham, MA), while the neutrophils were labelled by anti-Ly6G-488 antibody (0.1 mg/kg, Biolegend, cat#: 127,626, San Diego, CA), which were administered in the form of mixed reagent (100 μl) via retroorbital vein injection 15 min prior to the two-photon imaging. For image acquisition, a two-photon microscope (Olympus FV31S) equipped with a 25 × water immersion objective and a laser wavelength of 920 nm were utilized. Time-series images of the cerebral cortex were captured at a depth of 100 μm below the leptomeninges. The scanning resolution of images was set at 1024 × 1024 pixels, with a scanning thickness of 30 μm and a 2-μm interval between scans. The total scanning time was 30 min, with each time-lapse sequence lasting approximately 1 min. Only vessels with diameter less than 10 μm were included to restrict analysis to capillaries. The number of stalled neutrophils in the capillaries within the visual field was counted over a 30-min period, and the percentage of stalled neutrophils per square millimeter was calculated.

### Evans blue assay

Evans blue assay was performed according to previous studies [42, 43]. Briefly, one month after bevacizumab treatment, 4 ml/kg of 2% Evans blue in 0.9% saline was administered to mice by orbital intravenous injection. An hour later, the mice were anaesthetized and blood was collected. Mice were then transcardially perfused with 0.9% saline and the brains were dissected and homogenized. Brain homogenates or plasma was combined with 60% trichloroacetic acid at a ratio of 6:5. Absorbance at 620 nm was determined using a microplate reader (TECAN, Männedorf, Switzerland), and dye concentration was calculated using a standard curve. Dye content in the brain was normalized to the weight of homogenized tissue and the dye content in the plasma.

### CBF test

The mice were anesthetized by sodium pentobarbital (100 mg/kg body weight), and CBF was recorded after they reached continuous and stable breathing [44, 45]. The scalp and periosteum were removed. The skull was exposed, wiped clean, and kept moist with 0.9% saline. The tissue blood flow scanning imaging analysis system (PERIMED PSI-ZR, Sweden) was placed on top of the mouse skull to monitor and record the cerebral blood perfusion of neocortex before and after intraperitoneal injection of norepinephrine (0.2 mg/ml in 0.9% saline, 100 µl). The average CBF perfusion value of mice before norepinephrine administration was used as baseline. Changes in CBF were averaged and expressed as percent (%) increase of baseline.

### Immunofluorescence staining

After completion of the behavioral tests, mice were anesthetized by sodium pentobarbital (100 mg/kg body weight). Surgical scissors were used to expose the heart, followed by transcardial perfusion of PBS for 2 min and PBS with 4% paraformaldehyde for an additional 5 min at a rate of 10 ml/min. The brains were harvested and post-fixed in PBS with 4% paraformaldehyde at 4 °C overnight, followed by cryoprotection in 30% sucrose in PBS for 2 to 3 days at 4 °C. Then 30-μm coronal sections were cut by a cryostat for free-floating immunofluorescence staining. The dorsal hippocampus (dHC) and the primary sensory cortex (S1) at approximately 1.58 mm and 1.82 mm posterior to the Bregma were sampled for each staining. The sections were incubated in the blocking buffer (5% normal goat serum, 1% bovine serum albumin in PBS with 0.4% Triton X-100) for 2 h at room temperature, and stained with primary antibodies diluted in the blocking buffer for approximately 40 h at 4 °C. The primary antibodies used in this study included: anti-GFAP (Abcam, cat#: ab4674, 1:5000, Cambridge, United Kingdom), anti-Aβ42 (BioLegend, cat#: 803001, 1:2000), anti-CD31 (BD-biosciences, cat#: 550274, 1:100, San Jose, CA), and anti-IBA1 (Fujifilm, cat#: 019-19741, 1:500, Richmond, VA). After 3 × 10 min washes in PBS with 0.4% TritonX-100, the sections were stained with secondary antibodies diluted in the blocking buffer for 2 h at room temperature, followed by additional washes before mounting onto glass-slides. The secondary antibodies used in this study included: goat anti-mouse IgG(H + L) Alexa Fluor Plus647 (Thermo Fisher, cat#: A21236, 1:1000), goat anti-rabbit IgG(H + L) Alexa Fluor Plus568 (Thermo Fisher, cat#: A11036, 1:1000), goat anti-chicken IgY(H + L) Alexa Fluor Plus488 (Thermo Fisher, cat#: A32931, 1:1000), and goat anti-rat IgY(H + L) Alexa Fluor Plus650 (Thermo Fisher, cat#: SA5-10021, 1:1000). Images were taken by an epi-fluorescent microscope (Nikon Eclipse Ni-U, Tokyo, Japan), and analyzed by ImageJ (version 1.52p, NIH, Rockville, MD).

### Blood pressure test

The blood pressure and heart rate of mice were monitored using Softron (BP-2010A, Beijing, China). Blood pressure was measured non-invasively by the tail pressure photoelectric volume pulse method [46]. During the measurement, the mice were tested in a nonanesthetized state, placed in the mouse net and kept at a constant temperature. The sensor was placed at the base of the mouse’s tail to measure blood pressure.

### Measurement of brain and plasma VEGF levels

Mouse brain tissues were homogenized in ice-cold RIPA lysis buffer (1 × PBS buffer containing 1% NP-40, 0.5% Na deoxycholate, and 0.1% SDS) with a protease & phosphatase inhibitor mixture (Thermo Fisher, cat#: UG280144) [47]. Orbital sinus blood was collected under anesthesia. For each mouse, ~ 100 μl of whole blood was collected into a 0.5-ml tube coated with anticoagulant (Kang Jian Medicale, 044–0241). The samples were centrifuged at 4 °C for 15 min at 2500 rpm. The supernatants were collected as plasma and stored at − 80 °C until analysis. Plasma and brain VEGF concentrations were measured with an enzyme-linked immunosorbent assay (ELISA) kit (NeoBioscience, EMC103.96, Shenzhen, China) following the manufacturer’s instructions.

### Immunoblotting analysis

Mouse brain tissues were homogenized in ice-cold RIPA lysis buffer (1 × PBS buffer containing 1% NP-40, 0.5% Na deoxycholate, and 0.1% SDS) with a protease & phosphatase inhibitor cocktail (Thermo Fisher, cat#: UG280144) [47]. Protein concentrations were determined using the BCA protein detection kit (Thermo Fisher, cat#: 23227), and equal amounts of protein (10 µg per lane for tissue lysates) were resolved on denaturing 10% SDS–PAGE gels and transferred by electroblotting to PVDF membranes (Millipore, cat#: IPVH00010, Burlington, MA). Membranes were incubated with anti-VEGFR1 (Abcam, cat#: ab2350, 1:500), anti-VEGFR2 (Abcam, cat#: ab221679, 1:1000), anti-Cgn (Sigma, cat#: HPA027657, 1:500, St. Louis, MO), anti-ZO-1 (Invitrogen, cat#: 61–7300, 1:1000, Carlsbad, CA), anti-Claudin 5 (Invitrogen, cat#: 34–1600, 1:1000), or anti-Actin (Millipore, cat#: MAB1501R, 1:5000). The membranes were washed with PBST (0.2% Tween-20 in PBS), incubated with a horseradish peroxidase (HRP)-conjugated goat anti-mouse IgG (Earthox, cat#: E030110-01, 1:20,000, Millbrae, CA) or HRP goat anti-rabbit IgG (Earthox, cat#: E030120-01, 1:20,000) for 1 h, washed again, and incubated with ECL detection reagents (Millipore, cat#: WBKLS0500). Densitometry analysis was performed using the ImageJ (version 1.52p, NIH) software.

### Tissue dissection, RNA extraction and RNA sequencing

Mice were sacrificed after one month of bevacizumab treatment. The brains were sliced into 1-mm sections on a brain matrix (RWD Life Science, Shenzhen, China) in ice-cold dissection buffer (2.6 mM KCl, 1.23 mM NaH_2_PO_4_, 26.2 mM NaHCO_3_, 5 mM kynurenic acid, 212.7 mM sucrose, 10 mM dextrose, 0.5 mM CaCl_2_, 1 mM MgCl_2_). The hippocampus and cortex were dissected out and snap-frozen on dry ice. Total RNA was extracted using Trizol (Thermo Fisher, cat#: 15596018). The RNA samples were submitted to the GENEWIZ (Suzhou, China) for quality control using Agilent TapeStation 2200. All RNA samples had RNA integrity numbers > 8. The samples further underwent library construction and sequenced by an Illumina NovaSeq 6000 system for paired-end 150 bp reads.

### Analyses of RNA sequencing results

The hippocampal RNA was extracted from vehicle-treated wild-type, vehicle-treated 5×FAD, and bevacizumab-treated 5×FAD mice of both sexes. RNA-sequencing (RNA-seq) was performed on three independent biological pools of samples (2–3 animals were mixed as one biological pool) from each sex. Raw data of fastq format were processed by Trimmomatic (version 0.36) to acquire the clean reads, which were then mapped to NCBI Rfam databases, to remove the rRNA sequences by Bowtie2 (version 2.33). The reads were mapped to the mouse reference genome using the Hisat2 (version 2.1.0) [48, 49]. HTSeq-count (version 0.9.1) was used to obtain the read count and function information of each gene. The count tables were normalized based on their library size using trimmed mean of M-values (TMM) normalization implemented in R/Bioconductor EdgeR (version 3.34.0) [50, 51]. Normalized read counts were fitted to a negative binomial distribution with a quasi-likelihood F-test. Principal component analysis (PCA) was performed for the regularized log transform (rlog) of the normalized counts using plotPCA tools with default parameters [52, 53]. Differential gene expression analysis was further carried out using EdgeR. The transcripts were considered as differentially expressed genes (DEGs) at *P* value < 0.05 with Benjamini–Hochberg correction for multiple testing. Volcano plots, Venn plot and heatmaps were generated using VennDiagram (version 1.6.20), ggplot2 (version 3.4.2), and pheatmap (version 1.0.12) packages in R/Bioconductor [50].

To identify cell type-enriched transcripts, we compared our DEGs to a database of cell type-specific mRNA expression published by Zhang et al. [54] which established selectively enriched transcripts in neurons, glia, and vascular cells of mouse cerebral cortex. Using fragments per kilobase of transcript per million mapped reads (FPKM) numbers for astrocytes, endothelial cells, neurons, microglia, and oligodendrocytes, we calculated the enrichment scores of the transcripts as follows: enrichment score in cell type X = FPKM of transcripts expressed in cell type X/FPKM of transcripts expressed in all other cell types. The DEGs with enrichment scores > 1.5 in a given cell type were considered as cell-type enriched.

### Validation of RNA sequencing data by quantitative PCR (qPCR)

qPCR primers were selected from the PrimerBank (https://pga.mgh.harvard.edu/primerbank/) or designed using Primer-BLAST (https://www.ncbi.nlm.nih.gov/tools/primer-blast/). The specificity of the primers was further confirmed with BLAST (https://blast.ncbi.nlm.nih.gov/Blast.cgi) and melting curve analysis, and the amplification efficiency of the primers was examined by qPCR using serial dilutions of a cDNA template. The sequences of the primers are listed in Additional file 1: Table S1. The cDNAs were synthesized using the NovoScript Plus All-in-on Strand cDNA Synthesis Supermix (Novoprotein, Suzhou, China) following the manufacturer’s instructions. qPCR was performed using a CFX96 Touch Real-Time PCR Detection System (Bio-Rad) with the NovoStart SYBR qPCR SuperMix Plus (Novoprotein). For each sample, the cDNA was amplified using one initial denaturation step at 95 °C for 1 min, followed by 40 cycles of 95 °C for 20 s, 60 °C for 20 s, and 72 °C for 30 s. Triplicates of each sample were analyzed by qPCR, and the mean cycle quantification (Cq) value was used for calculating the relative expression of target mRNAs using the ΔΔCt method. *Gapdh *served as the internal control.

### Gene set enrichment analysis (GSEA)

GSEA (Broad Institute, version 4.3.2) was performed to identify changes in functional enrichment of the transcriptomic profiles, using the gene set databases of Gene Ontology (GO, c5.go.bp.v7.5.1.symbols.gmt, c5.go.mf.v7.5.1.symbols.gmt, and c5.go.cc.v7.5.1.symbols.gmt) and Kyoto Encyclopedia of Genes and Genomes (KEGG) pathways (c2.cp.kegg.v7.5.1.symbols.gmt). Gene set size filters were set at minimum of 5 and maximum of 1000. False discovery rate (FDR) for the enrichment score of the gene set was calculated based on 1000 gene set permutations. The top gene sets enriched in each group were plotted with ggplot2 (version 3.4.2) in R.

### Statistical analyses

Data are presented as mean ± SEM. Statistical analyses were carried out using GraphPad Prism (version 8, GraphPad Software, Boston, MA). The Shapiro–Wilk test was used to evaluate the normality of the datasets. An outlier test with the ROUT method was applied to identify potential outliers in the datasets. For two-group comparisons, an unpaired two-tailed Student’s* t* test was used for normally distributed datasets. For multiple-group comparisons, one-way ANOVA followed by Fisher’s LSD test and two-way ANOVA followed by Tukey’s multiple comparison test were used for normally distributed datasets, and the Kruskal–Wallis test followed by Dunn’s test was used for nonnormally distributed datasets. *P* < 0.05 was considered statistically significant.

## Results

### Bevacizumab treatment improves long-term memory in both sexes of 5×FAD mice

**Fig. 1:**
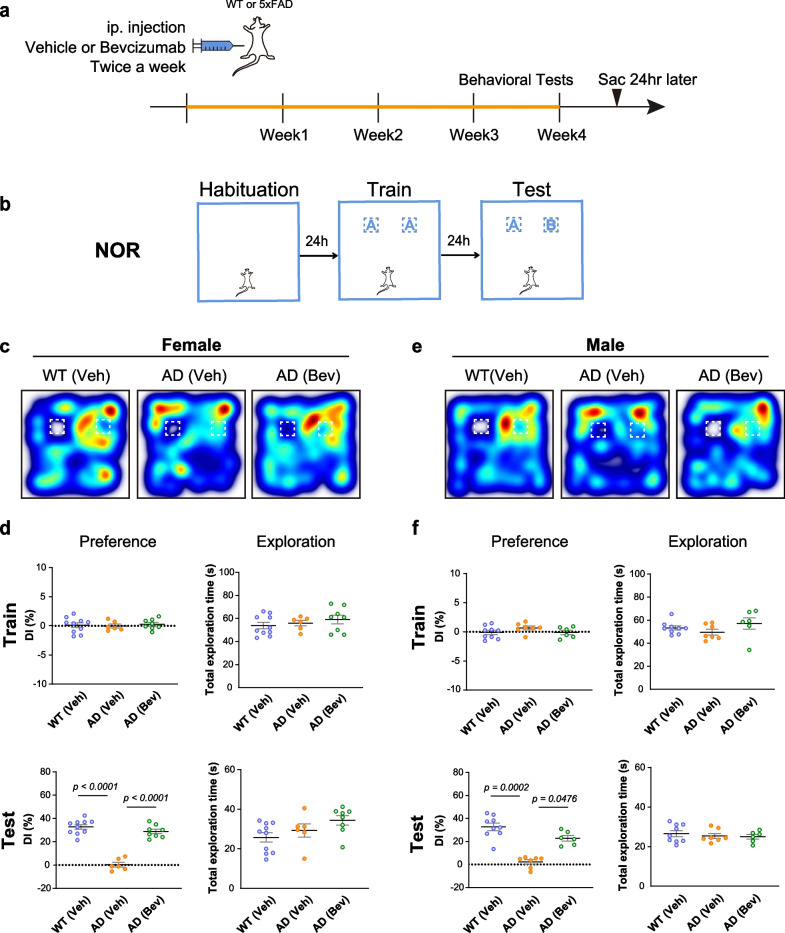
Bevacizumab treatment improves long-term memory in 5×FAD mice. **a** The experimental timeline of bevacizumab treatment. **b** Schematic of the novel object recognition (NOR) task. A and B indicate different objects. **c** Representative heatmaps of the traveling paths of female mice during the test session of the NOR task. **d** The discrimination index (DI%) and the total time spent exploring both objects (total exploration time) during the training and test sessions in the NOR task (*n* = 6–10 female mice per group).** e** Representative heatmaps of the traveling paths of male mice during the test session of the NOR task.** f** The discrimination index (DI%) and the total time spent exploring both objects (total exploration time) during the training and test sessions in the NOR task (*n* = 6–9 male mice per group). Data are presented as the mean ± SEM and were analyzed by one-way ANOVA followed by Fisher’s LSD test, except Test DI (%) data in panel **f**, which were analyzed by the Kruskal–Wallis test followed by Dunn’s test. WT (Veh): wild-type littermates receiving sham treatment, AD (Veh): 5×FAD mice receiving sham treatment, AD (Bev): 5×FAD mice receiving bevacizumab treatment

The roles of VEGF in cognitive impairment during the early stage of AD pathological development have not been examined previously. We therefore administered bevacizumab (10 mg/kg) to 4-month-old 5×FAD mice and wild-type littermates of both sexes by intraperitoneal injection twice a week for one month [35]. At this age, amyloid deposits in the hippocampus and memory deficits begin to occur in 5×FAD mice [30, 32]. During the last week of treatment, behavioral tests, including the open field test (on the first day of the three-day habituation) and the NOR test, were performed (Fig. 1a, b). The gross view of bevacizumab-treated mice was undistinguishable from that of saline-treated animals. There was no detectable difference in body weights among the bevacizumab-injected 5×FAD, vehicle-injected 5×FAD, and vehicle-injected WT mice after 1 month of treatment. The 5×FAD and wild-type mice exhibited no difference in the training stage of the NOR task, as demonstrated by similar exploration time on the training objects. Twenty-four hours after training, mice were tested for their preference for the novel object as an indicator of memory strength to the familiar object. Compared to the wild-type littermates, both sexes of 5×FAD mice injected with vehicle showed significant deficits in recognizing familiar objects and spent equal time interacting with both objects. Bevacizumab treatment rescued the object memory of 5×FAD mice to a similar level as the wild-type littermates (Fig. 1c–f). Bevacizumab treatment also showed an anxiolytic effect in female 5×FAD mice as demonstrated by increased exploration time in the center zone of the open arena (Additional file 1: Fig. S1a, b). However, no effects on the anxiety level of male 5×FAD mice were observed after bevacizumab treatment (Additional file 1: Fig. S1c, d).

Our findings indicated for the first time that specifically blocking VEGF by bevacizumab treatment in the early stage of amyloidopathy had protective effects on  long-term memory in both sexes of 5×FAD mice and an anxiolytic effect in female 5×FAD mice.

### Bevacizumab treatment reduces capillary stalling by neutrophil adhesion, and improves BBB integrity and the cerebrovascular response of 5×FAD mice

**Fig. 2:**
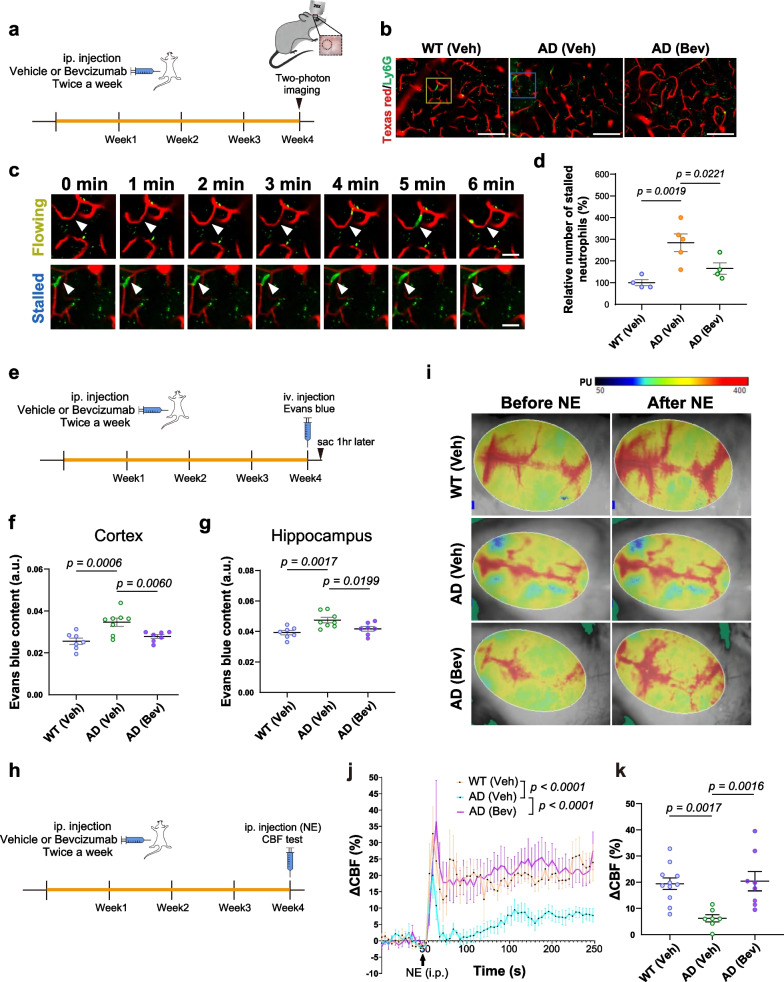
Bevacizumab treatment reduces capillary stalling of neutrophils and improves cerebrovascular responses to stress hormones in both sexes of 5×FAD mice. **a** Flow chart of the experimental design of in vivo two-photon imaging. Imaging was conducted through a cranial window in the cortex of anesthetized mice. **b** Representative two-photon projection images showing anti-Ly6G-488 antibody-labeled neutrophils trapped in capillaries. The vascular network was labeled with 70 kDa Texas Red dextran. Two-photon imaging was performed 15 min after the injection of both the anti-Ly6G-488 antibody and Texas Red dextran. Scale bars: 100 μm. **c** Enlarged images showing flowing or stalled neutrophils in cortical capillaries (red: Texas Red dextran-labeled blood vessels; Green: neutrophils labeled with anti-Ly6G-488). Scale bars: 25 μm. **d** The percentage of neutrophil stalls in capillaries was measured in both WT and 5×FAD mice (*n* = 4–5 mice per group, male and female combined). **e** Flow chart of the experimental design for Evans blue injection to determine blood–brain barrier integrity in 5×FAD mice. **f**, **g** Quantitative analysis of Evans blue dye leakage in the (**f**) cortex and (**g**) hippocampus of both male and female 5×FAD mice. Evans blue content was normalized by the weight of the cortex or hippocampus, followed by normalization to the plasma concentration of Evans blue (*n* = 7–8 mice per group, male and female mice combined). Data in** d**, **f** and **g** were analyzed by one-way ANOVA followed by Fisher’s LSD test. **h** Flow chart of the experimental design for the CBF test in 5×FAD mice. **i** Representative pseudocolor laser speckle flowmetry maps of CBF before and after norepinephrine injection into 5×FAD mice. **j** The curve graph shows the dynamic CBF changes (relative to the baseline CBF, ΔCBF) before and after norepinephrine injection (*n* = 7–11 mice per group). CBF changes in the WT (Veh), AD (Veh) and AD (Bev) groups at all time points were analyzed by two-way ANOVA followed by Tukey’s multiple comparison test. **k** Quantitative analysis of CBF changes relative to baseline CBF in response to norepinephrine injection in 5×FAD mice (*n* = 7–11 mice per group). The sum of CBF changes from the 48 s time point (immediately before norepinephrine injection) to the 248 s time point was averaged to represent the CBF changes in each animal, and then the results were analyzed by one-way ANOVA followed by Fisher’s LSD test. Data in (**d**), (**f**, **g**), and (**j**, **k**) showed similar trends of changes and effects by bevacizumab treatment in both female and male 5×FAD mice, and the data of both sexes were combined for statistical analysis. Data are presented as the mean ± SEM. CBF: cerebral blood flow, PU: units of blood perfusion. WT (Veh): wild-type littermates receiving sham treatment, AD (Veh): 5×FAD mice receiving sham treatment, AD (Bev): 5×FAD mice receiving bevacizumab treatment

Capillary stalling by increased neutrophil adhesion has been observed in the cortical vasculature of AD mouse models, and VEGF signaling is suggested to be involved in capillary stalling and cerebral perfusion reduction [11, 41, 55]. To examine whether bevacizumab treatment affects capillary stalling in 5×FAD mice, we performed a two-photon experiment through a cranial window to directly monitor the cortical vasculature (Fig. 2a). Consistent with a previous report, our results showed a significant increase in cortical stalling by neutrophil adhesion in both female and male 5×FAD mice at 5 months of age (Fig. 2a–d) [41]. Importantly, one month of bevacizumab treatment significantly reduced the number of neutrophils adhering to brain capillaries (Fig. 2d). Increased BBB leakage and reduced CBF have also been reported in 5×FAD mice at different ages [43, 56], but whether VEGF function is involved in vasculopathy in the early stage of AD remains undetermined. To address this question, we examined BBB leakage by orbital injection of Evans blue to monitor cerebrovascular leakage in 5-month-old 5×FAD mice of both sexes (Fig. 2e). The levels of Evans blue were increased in both cortical and hippocampal homogenates of 5×FAD mice compared with wild-type littermates (Fig. 2f, g; Additional file 1: Fig. S2a). Bevacizumab treatment effectively restored BBB integrity, as shown by the reduced Evans blue leakage in the cortex and hippocampus of both male and female 5×FAD mice (Fig. 2f, g; Additional file 1: Fig. S2a).

Changes in cerebral blood perfusion in response to cognitive performance have been considered to play critical roles in the metabolic support of adequate brain functions [57]. Notably, decreased cerebral blood perfusion has been shown to be a pathological feature in both AD patients and mouse models [15, 58–61]. Among the factors that can regulate cerebral blood perfusion, norepinephrine has been reported to have a vasoconstriction effect on cerebral vasculature and its administration can induce changes in regional or global CBF [62]. Norepinephrine is also known for its neurotransmitter roles in modulating neural activity and in a variety of cognitive functions and behavioral responses [63]. Because the locus coeruleus supplies norepinephrine to the brain and is among the brain regions first affected in AD pathology, a reduction in norepinephrine has long been considered to contribute to the development of cognitive dysfunctions in AD [63–65]. However, whether the cerebral vascular system of AD may respond to norepinephrine differently from the nondiseased brains has yet to be determined. To address this question, we monitored changes in the CBF of 5×FAD mice and wild-type littermates with intraperitoneal injection of norepinephrine. In agreement with previous reports [66, 67], we observed an acute elevation in CBF in wild-type mice after receiving norepinephrine injection. However, a significantly lower magnitude of the CBF response to norepinephrine was observed in both female and male 5×FAD mice compared with wild-type mice (Fig. 2h-k, Additional file 1: Fig. S2b). Importantly, we found that bevacizumab treatment effectively restored the CBF response to norepinephrine in 5×FAD mice to a level similar to that in wild-type mice (Fig. 2h–k, Additional file 1: Fig. S2b).

Of note, there was no significant difference in the density of blood vessels in the hippocampus and the S1 cortex among the wild-type, 5×FAD, and bevacizumab-treated 5×FAD mice, as detected by CD31 staining (Additional file 1: Fig. S3a, b). Furthermore, previous studies have reported that prolonged bevacizumab treatment may lead to hypertension in cancer patients [68, 69]. In our study, no differences in the blood pressure and heart rate were found among all groups after one month of bevacizumab treatment (Additional file 1: Fig. S4a–h).

Taken together, our findings suggest that blocking VEGF for one month by bevacizumab in 5×FAD mice of both sexes at early age improves BBB integrity and cerebrovascular perfusion in response to norepinephrine, without detectable changes in blood pressure, heart rate and cerebral vascular density.

### Bevacizumab treatment ameliorates Aβ deposition and glial activation in both sexes of 5×FAD mice

**Fig. 3:**
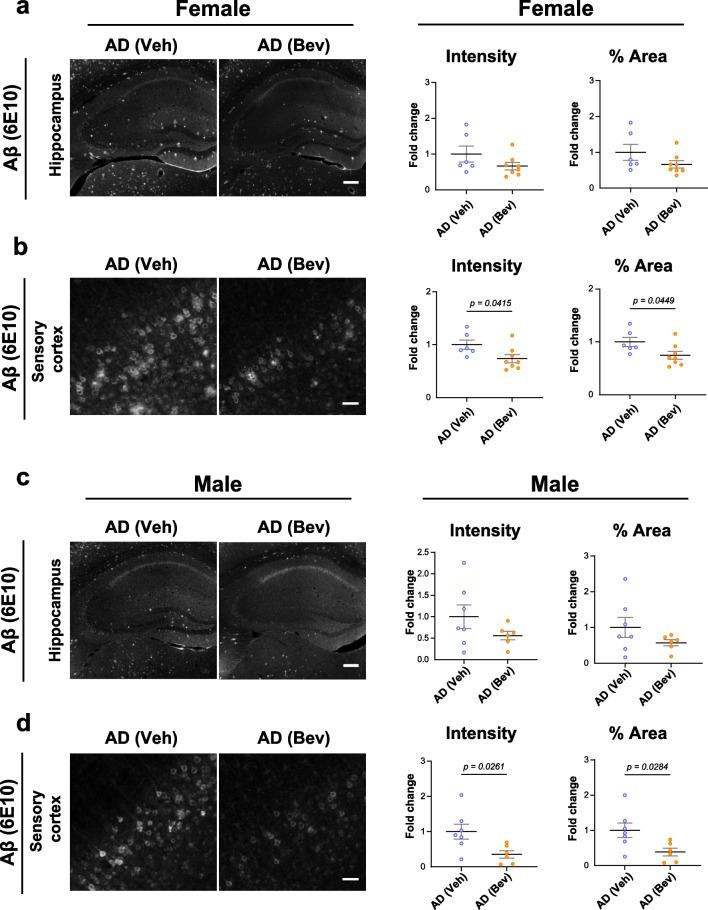
Bevacizumab treatment decreases Aβ levels in the brains of 5×FAD mice. **a** Representative images of Aβ immunofluorescence staining and quantitative analyses of Aβ fluorescence intensity and % area in the hippocampus of female mice (*n* = 6–8 mice per group). Scale bar: 200 µm. **b** Representative images of Aβ immunofluorescence staining and quantitative analyses of Aβ fluorescence intensity and % area in the sensory cortex of female mice (*n* = 6–8 mice per group). Scale bar: 50 µm. **c** Representative images of Aβ immunofluorescence staining and quantitative analyses of Aβ fluorescence intensity and % area in the hippocampus of male mice (*n* = 6–7 mice per group). Scale bar: 200 µm. **d** Representative images of Aβ immunofluorescence staining and quantitative analyses of Aβ fluorescence intensity and % area in the sensory cortex of male mice (*n* = 6–7 mice per group). Scale bar: 50 µm. All data are presented as the mean ± SEM and were analyzed by unpaired two-tailed Student’s *t* test. WT (Veh): wild-type littermates receiving sham treatment, AD (Veh): 5×FAD mice receiving sham treatment, AD (Bev): 5×FAD mice receiving bevacizumab treatment

Amyloid deposition is a pathological hallmark of AD. We examined the dHC, as its dysfunction is closely associated with memory deficits during AD progression [70, 71]. At 5 months of age, low to moderate Aβ deposition in the dHC of 5×FAD mice was detected by staining with anti-Aβ (6E10) antibody (Fig. 3a, c). We also included the primary sensory cortex (S1 cortex) in our analysis since more robust Aβ deposition was detected in the deep layers of the S1 cortex of 5×FAD mice [32] (Fig. 3b, d). After blocking VEGF by bevacizumab treatment, we observed a mild but nonsignificant reduction in Aβ deposition in the dHC of 5×FAD mice, likely due to the already low Aβ deposition levels at the age examined (Fig. 3a, c). Notably, a significant reduction in Aβ deposition was observed in the S1 cortex of both female and male 5×FAD mice (Fig. 3b, d).

**Fig. 4:**
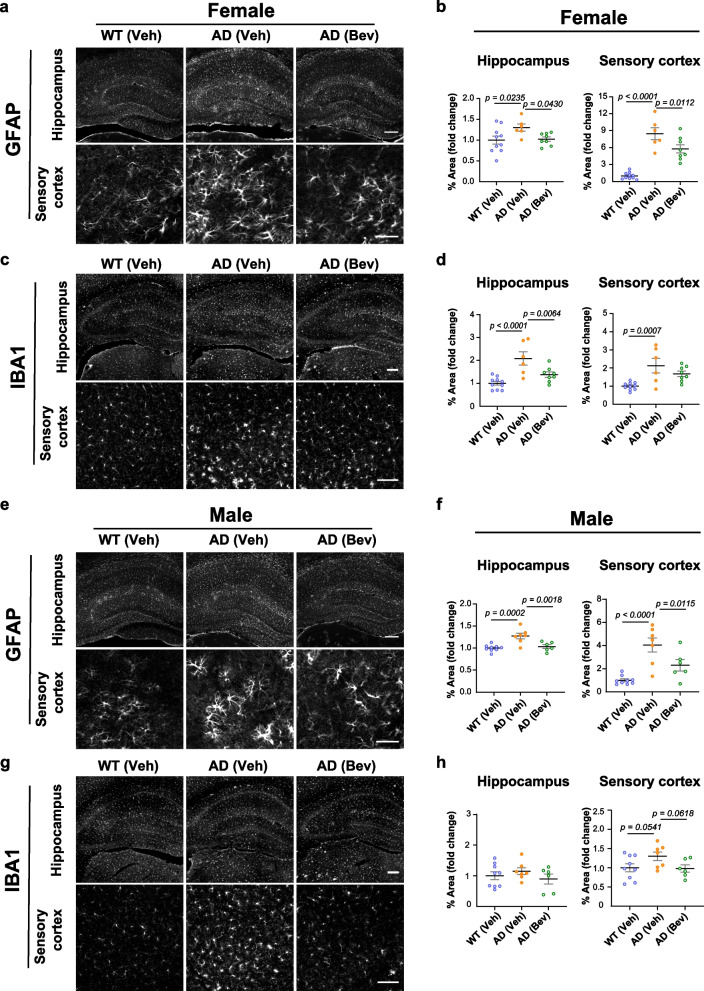
Reduced activation of glial cells in the brains of 5×FAD mice after bevacizumab treatment. **a** Representative images of GFAP immunofluorescence staining in the hippocampus and sensory cortex of female mice. Scale bar of the hippocampus: 200 µm. Scale bar of the sensory cortex: 50 µm. **b** Quantitative analyses of GFAP fluorescence % area in the hippocampus and sensory cortex of female mice (*n* = 6–10 mice per group). **c** Representative images of IBA1 immunofluorescence staining in the hippocampus and sensory cortex of female mice. Scale bar of the hippocampus: 200 µm. Scale bar of the sensory cortex: 100 µm. **d** Quantitative analyses of IBA1 fluorescence % area in the hippocampus and sensory cortex of female mice (*n* = 6–10 mice per group). **e** Representative images of GFAP immunofluorescence staining in the hippocampus and sensory cortex of male mice. Scale bar of the hippocampus: 200 µm. Scale bar of the sensory cortex: 50 µm. **f** Quantitative analyses of GFAP fluorescence % area in the hippocampus and sensory cortex of male mice (*n* = 6–9 mice per group). **g** Representative images of IBA1 immunofluorescence staining in the hippocampus and sensory cortex of male mice. Scale bar of the hippocampus: 200 µm. Scale bar of the sensory cortex: 100 µm. **h** Quantitative analyses of IBA1 fluorescence % area in the hippocampus and sensory cortex of male mice (*n* = 6–9 mice per group). All data are presented as the mean ± SEM and were analyzed by one-way ANOVA followed by Fisher’s LSD test. WT (Veh): wild-type littermates receiving sham treatment, AD (Veh): 5×FAD mice receiving sham treatment, AD (Bev): 5×FAD mice receiving bevacizumab treatment

Associated with BBB leakage and Aβ deposition, the activation of astrocytes and microglia plays critical roles in the pathogenesis of AD [72–75]. As bevacizumab treatment reduced BBB leakage and Aβ deposits in the hippocampus and the cortex of 5×FAD mice, we further examined whether such changes are accompanied by altered activation of microglia and astrocytes. Consistent with previous reports, increased expression of glial fibrillary acidic protein (GFAP) in astrocytes and ionized calcium-binding adapter molecule 1 (IBA1) in microglia, which are indicative of astrocytic and microglial activation, was observed in the dHC and the S1 cortex of 5×FAD mice at 5 months of age (Fig. 4). The only exception was the male dHC which showed no increase in IBA1 (Fig. 4g, h). Bevacizumab treatment effectively reduced GFAP expression levels in the dHC and S1 cortex of both sexes of 5×FAD mice (Fig. 4), while IBA1 expression in the female dHC and male S1 cortex of 5×FAD mice was also reduced after bevacizumab treatment.

Collectively, these data suggest that anti-VEGF treatment by bevacizumab alleviated the pathological development of Aβ deposits as well as astrocytic and microglial activation in the early stage of 5×FAD mice.

### Sex-specific changes in the levels of soluble VEGFR1 (sVEGFR1) and full-length VEGFR2 proteins in 5×FAD mice

Previous studies have reported inconsistent results for VEGF protein levels in the brains and peripheral blood of AD patients and mouse models [23, 25, 26, 76–79]. To examine whether the differences in VEGF protein levels exist in 5×FAD mice at 5 months of age, we collected the hippocampus, S1 cortex, and plasma for ELISA detection of VEGF protein (Additional file 1: Fig. S5a, b). No detectable changes in VEGF protein level between the 5×FAD mice and wild-type littermates in either sex were observed. Bevacizumab treatment significantly reduced hippocampal VEGF levels in male but not female 5×FAD mice (Additional file 1: Fig. S5a, b).

As the main receptor that mediates VEGF function, VEGFR2 protein has been reported to be upregulated in the cortex of APP/PS1 and 5×FAD mice at 10–12 months of age [11, 23, 79, 80]. Considered as a repressor of VEGF function in angiogenesis, VEGFR1 protein levels have been  reported to be decreased in the parietal cortex of AD patients and in the cortex of 10- to 11-month-old male APP/PS1 mice [11, 23, 79]. sVEGFR1 is a truncated form of VEGFR1 that lacks a transmembrane domain; it has been considered as a negative regulator of angiogenesis by sequestering VEGF and antagonizing VEGFR2 function [23]. It is yet to be determined whether altered expression levels of sVEGFR1, VEGFR1 and VEGFR2 are associated with BBB leakage and impaired cerebrovascular response in 5×FAD mice at 4–5 months of age.

**Fig. 5:**
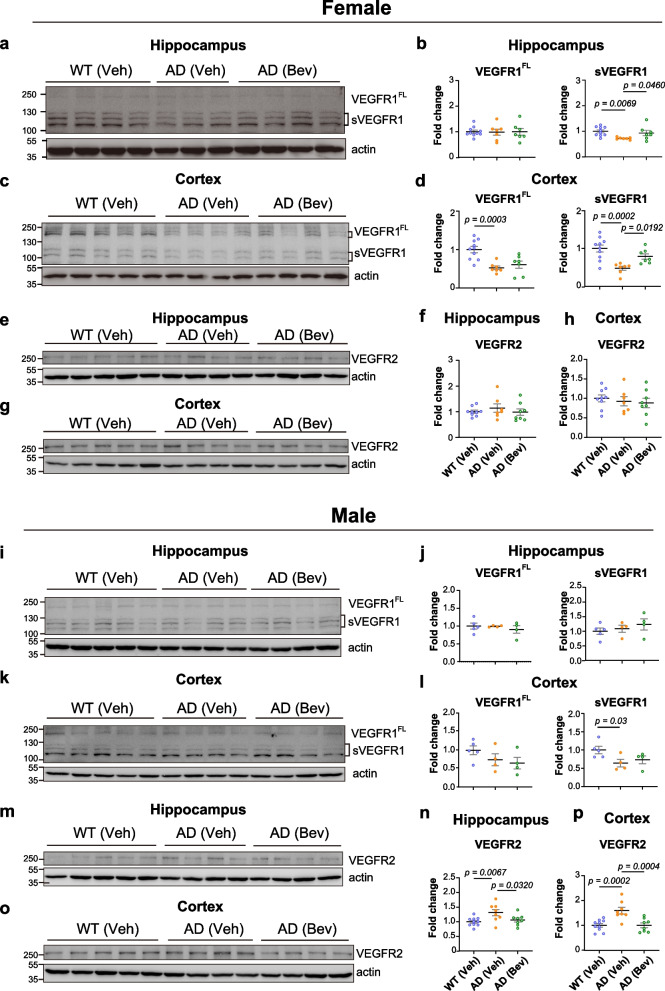
Sex-specific changes in the levels of VEGF receptors in the 5×FAD mouse brain. (**a**) Representative images and (**b**) quantitative analysis of full-length VEGFR1 (VEGFR1^FL^) and soluble VEGFR1 (sVEGFR1) protein levels in the hippocampus of female 5×FAD mice (*n* = 7–10 mice per group). (**c**) Representative images and (**d**) quantitative analysis of VEGFR1^FL^ and sVEGFR1 protein levels in the cortex of female 5×FAD mice (*n* = 7–10 mice per group). (**e**) Representative images and (**f**) quantitative analysis of VEGFR2 protein levels in the hippocampus of female 5×FAD mice (*n* = 7–9 mice per group). (**g**) Representative images and (**h**) quantitative analysis of VEGFR2 protein levels in the cortex of female 5×FAD mice (*n* = 7–9 mice per group). (**i**) Representative images and (**j**) quantitative analysis of VEGFR1^FL^ and sVEGFR1 protein levels in the hippocampus of male 5×FAD mice (*n* = 4–5 mice per group). (**k**) Representative images and (**l**) quantitative analysis of VEGFR1^FL^ and sVEGFR1 protein levels in the cortex of male 5×FAD mice (*n* = 4–5 mice per group). (**m**) Representative images and (**n**) quantitative analysis of VEGFR2 protein levels in the hippocampus of male 5×FAD mice (*n* = 8–10 mice per group). (**o**) Representative images and (**p**) quantitative analysis of VEGFR2 protein levels in the cortex of male 5×FAD mice (*n* = 8–10 mice per group). All data are presented as the mean ± SEM and were analyzed by one-way ANOVA followed by Fisher’s LSD test. WT (Veh): wild-type littermates receiving sham treatment, AD (Veh): 5×FAD mice receiving sham treatment, AD (Bev): 5×FAD mice receiving bevacizumab treatment

To directly examine the protein levels of VEGF receptors in the hippocampus and cortex of both sexes of 5×FAD mice, immunoblotting analysis was performed. Interestingly, sex differences in VEGFR expression were found in 5×FAD mice. In female mice, full-length VEGFR1 protein (VEGFR1^FL^) levels were decreased in the cortex but not in the hippocampus (Fig. 5a–d). We noticed a significant reduction in sVEGFR1 protein levels in both the hippocampus and cortex of female 5×FAD mice, which could be restored by bevacizumab treatment (Fig. 5a–d). Full-length VEGFR2 protein levels remained unaltered in the female 5×FAD mice compared with wild-type mice (Fig. 5e–h).

In male mice, the levels of full-length VEGFR1 protein remained unaltered compared with those in wild-type mice (Fig. 5i, j, l). A decrease of sVEGFR1 protein level was found in the cortex, which was not changed after bevacizumab treatment (Fig. 5k, l). In contrast, the protein levels of VEGFR2 were significantly increased in both the hippocampus and the cortex of male 5×FAD mice (Fig. 5m–p), and bevacizumab treatment significantly lowered them to levels comparable to those in wild-type mice (Fig. 5m–p).

Collectively, our findings unexpectedly revealed increased VEGF function through sex-specific alterations of two types of VEGF receptor in the early stage of amyloidopathology, and these alterations were alleviated by bevacizumab treatment.

### Bevacizumab treatment reverses the alterations of the hippocampal transcriptomic signatures associated with BBB integrity, cerebrovascular function, and neuronal activity

**Fig. 6:**
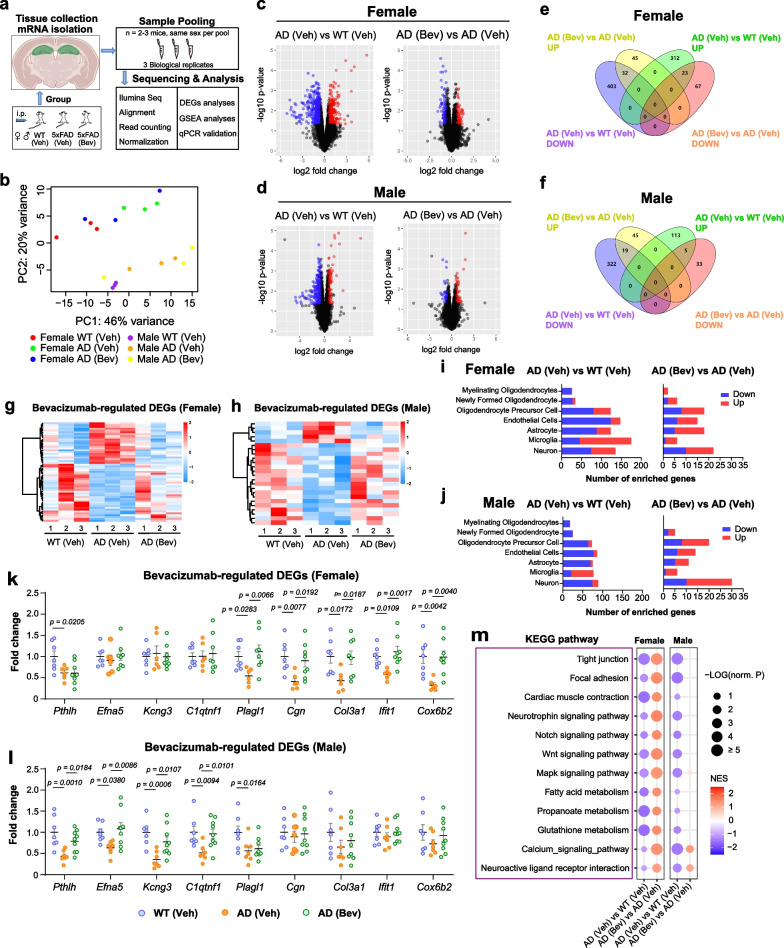
Reversal of transcriptomic profiles enriched in cerebrovascular and mitochondrial functions in bevacizumab-treated 5×FAD mice. **a** Experimental design and the workflow of the RNA-seq analysis. **b** Principal component analysis (PCA) plot of the RNA-seq results (*n *= 3 pools per group, 2–3 animals of the same sex per pool). **c** Volcano plots of the DEGs of the female groups, with horizontal lines at −log10 (*P* value) = −log10 (0.05) and vertical lines at log2 (fold change, FC) = log2 (1.5). **d** Volcano plots of the DEGs of the male groups. **e** Venn diagram depicting limited overlaps of the DEGs of the female groups. **f** Venn diagram depicting limited overlaps of the DEGs of the male groups. **g** Heatmaps of the DEGs affected by bevacizumab treatment in female 5×FAD mice. Twenty-three DEGs were upregulated in 5×FAD mice compared with wild-type mice but downregulated after bevacizumab treatment. Thirty-two DEGs were downregulated in 5×FAD mice but upregulated after bevacizumab treatment. **h** Heatmaps of the DEGs affected by bevacizumab treatment in male 5×FAD mice. Five DEGs were upregulated in 5×FAD mice but downregulated after bevacizumab treatment. Nineteen DEGs were downregulated in 5×FAD mice but upregulated after bevacizumab treatment. **i**, **j** Cell-type enrichment analysis of DEGs in the (**i**) female groups and (**j**) male groups. **k**, **l** Quantitative PCR validation of DEGs identified from 5×FAD versus wild-type groups, which were reversed by bevacizumab treatment in the (**k**) female (*n* = 6–8 mice per group) and (**l**) male (*n* = 7–9 mice per group) groups. All data are presented as the mean ± SEM and were analyzed by one-way ANOVA followed by Fisher’s LSD test. **m** KEGG pathway analysis based on GSEA was performed for RNA-seq data of the female and male 5×FAD mice. WT (Veh): wild-type littermates receiving sham treatment, AD (Veh): 5×FAD mice receiving sham treatment, AD (Bev): 5×FAD mice receiving bevacizumab treatment

To elucidate the molecular mechanisms underlying VEGF-mediated brain pathology in 5-month-old 5×FAD mice, hippocampal RNA was extracted from 5×FAD and wild-type mice after completion of the bevacizumab treatment. RNA-sequencing analysis was performed to identify differentially expressed genes (DEGs, *P* < 0.05 by edgeR) and clustered heatmaps were constructed (Fig. 6a–h and Additional file 1: Fig. S6a–d). More DEGs were identified in the female group than in the male group (Additional file 1: Fig. S6e). Cell-type specific enrichment analysis showed that the DEGs downregulated in the hippocampus of 5×FAD mice compared to the wild-type littermates were mostly enriched in endothelial cells, while the upregulated DEGs were mostly enriched in microglia (Fig. 6i, j). Moreover, bevacizumab treatment resulted in most changes in neuron-enriched DEGs in both female and male 5×FAD mice (Fig. 6i, j).

Nine DEGs downregulated in 5×FAD mice but reversed by bevacizumab treatment were validated by quantitative PCR (Fig. 6k, l). Six of the genes were functionally associated with angiogenesis, vessel permeability or vasoconstriction, including *Cgn* (cingulin) [81, 82], *Col3a1* (collagen, type III, alpha 1) [83, 84], and *Plagl1* (pleiomorphic adenoma gene-like 1) specifically in female mice [85], as well as *Pthlh* (parathyroid hormone-like peptide) [86, 87], *Efna5* (ephrin A5) [88, 89], and *Kcng3* (potassium voltage-gated channel, subfamily G, member 3) [90–92] specifically in male mice. These findings suggest that the protective effects of bevacizumab against cerebrovascular-related pathology in 5×FAD mice may be sex-specific. Other DEGs included *C1qtnf1* (C1q and tumor necrosis factor related protein 1), *Ifit1* (interferon-induced protein with tetratricopeptide repeats 1), and *Cox6b2* (cytochrome c oxidase subunit 6B2), which may be involved in disease progression due to their regulatory roles in inflammation, innate immunity, or mitochondria-associated metabolism [93–95].

To gain functional insights into the protective effects of bevacizumab in 5×FAD mice, GSEA was performed on the hippocampal RNA-seq data for KEGG pathways and GO enrichment [96]. Twelve gene sets were identified for each analysis (|NES|> 1, NOM *P* value < 0.05) (Fig. 6m, Additional file 1: Fig. S6f). Importantly, bevacizumab treatment restored gene sets in KEGG pathways that are functionally involved in the regulation of vascular integrity and functions (tight junction, focal adhesion, cardiac muscle contraction, Notch signaling, and Wnt signaling), metabolism (fatty acid metabolism, propanoate metabolism, and glutathione metabolism) and neurotrophin signaling pathways in the female 5×FAD mice. Additionally, bevacizumab restored gene sets that regulate neuronal functions (Mapk signaling pathway, calcium signaling pathway, and neuroactive ligand receptor interaction) in both female and male 5×FAD mice (Fig. 6m). The top enriched GO terms that were downregulated in female 5×FAD mice included gene sets associated with mitochondrial functions (mitochondrial respiratory chain complex assembly, mitochondrial protein-containing complex, respiratory chain complex, and electron transfer activity), regulation of translation (ribosomal subunit, and translation factor activity RNA binding), and ubiquitin-like protein ligase binding, and these pathways were restored in bevacizumab-treated 5×FAD mice (Additional file 1: Fig. S6f). Gene sets of pathways that regulate vascular functions (cell–cell adhesion via plasma membrane adhesion molecules and smooth muscle contraction), ncRNA-mediated regulation of translation, receptor complexes, and voltage-gated cation channel activity were downregulated in both female and male 5×FAD mice but restored after bevacizumab treatment (Additional file 1: Fig. S6f).

Notably, DEGs that were specifically altered by bevacizumab treatment but did not overlap with the 5×FAD DEGs, as shown in Fig. 6e and 6f, may also participate in the improved vascular and cognitive function of 5×FAD mice. To gain functional insight into these DEGs, we performed additional pathway enrichment analyses of these bevacizumab-specific DEGs (Additional file 1: Fig. S6g). The results showed sex-specific differences in the enriched functions of these bevacizumab-specific DEGs. In females, the DEGs were associated with the G protein-coupled serotonin receptor signaling pathway, T-cell differentiation, positive regulation of smooth muscle proliferation, myelination, and regulation of the receptor signaling pathway via JAK-STAT, while in males, the DEGs were associated with the acetylcholine receptor signaling pathway, regulation of cell adhesion mediated by integrin, synaptic vesicle exocytosis, calcium ion binding, and neuroactive ligand-receptor interaction (Additional file 1: Fig. S6h).

**Fig. 7:**
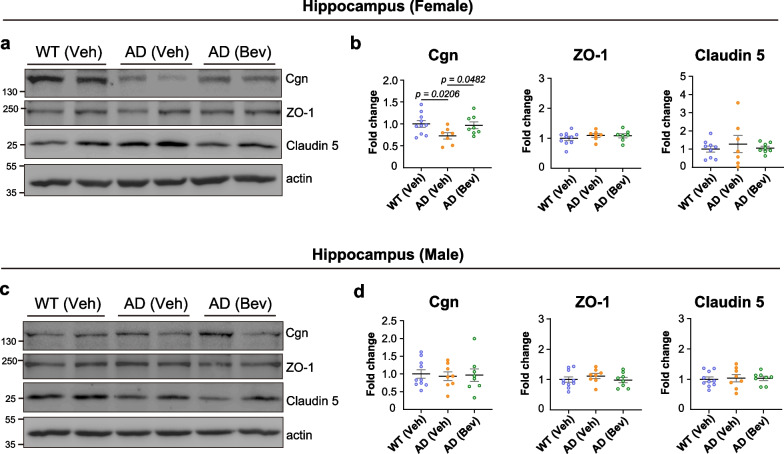
The levels of proteins associated with the blood–brain barrier in the hippocampus of 5×FAD mice. (**a**) Representative images and (**b**) quantitative analysis of Cgn, ZO-1, and claudin 5 protein levels in the hippocampus of female 5×FAD mice (*n* = 7–10 mice per group). (**c**) Representative images and (**d**) quantitative analysis of Cgn, ZO-1, and claudin 5 protein levels in the hippocampus of male 5×FAD mice (*n* = 8–10 mice per group). All data are presented as the mean ± SEM and were analyzed by one-way ANOVA followed by Fisher’s LSD test. WT (Veh): wild-type littermates receiving sham treatment, AD (Veh): 5×FAD mice receiving sham treatment, AD (Bev): 5×FAD mice receiving bevacizumab treatment. Cgn: cingulin, ZO-1: zonula occludens-1

To validate the key results of our RNA-seq findings at the protein level, immunoblotting analysis was performed for the BBB-associated DEGs. Consistent with the RNA-seq findings, decreased hippocampal cingulin protein level was observed in female but not male 5×FAD mice compared with their wild-type littermates, and bevacizumab treatment significantly increased the hippocampal cingulin protein level in female 5×FAD mice (Fig. 7). The protein levels of other endothelial tight junction proteins, including ZO-1 and claudin 5, were also examined. Unexpectedly, we found no significant changes in the protein levels of ZO-1 and claudin 5 in either sex of 5×FAD mice at the age of 5 months (Fig. 7).

Taken together, our findings reveal the molecular signatures underlying bevacizumab-mediated protection against amyloidosis-induced cerebrovascular pathology and memory deficits at the early stage of AD pathology in the 5×FAD mouse model.

## Discussion

In the current study, we identified that specific blockade of VEGF function by bevacizumab treatment effectively alleviated AD-related cerebrovascular pathology and restored long-term memory in 5×FAD mice in the early stage of disease progression. Importantly, our results revealed for the first time sex-specific differences in the levels of sVEGFR1 and full-length VEGFR2 proteins in the hippocampus and cortex of 5×FAD mice, and these levels were restored to levels similar to wild-type mice after bevacizumab treatment. These findings suggest that imbalanced VEGF signaling in the brain parenchyma of early amyloidopathy may contribute to the development of cerebrovascular dysfunction, which can be prevented by an anti-VEGF intervention strategy.

Neurovascular dysfunction plays critical roles in the pathogenesis of AD and other types of dementia [15, 97–99]. Regional blood supply in response to neural activity is critical for normal cognitive functions [98]. The degree of CBF reduction has been shown to correlate with the severity of cognitive dysfunction, suggesting that impaired CBF may directly contribute to cognitive decline in both the aged population and patients with AD [97, 100]. Increased BBB leakage has also been observed in the brains of both AD patients and mouse models, which is considered to affect cerebral blood supply and worsen neuroinflammation [43, 56, 101, 102]. Our findings demonstrate a significant increase in cortical stalling by neutrophil adhesion, an increase in the leakage of the BBB, and a decrease in the CBF response to norepinephrine treatment in both female and male 5×FAD mice at 5 months of age, changes that can be restored by VEGF blockade by bevacizumab. These findings indicate that amyloidopathy-induced alteration in the VEGF signaling pathway may be involved in capillary stalling, vessel leakage, and reduced cerebrovascular response at an early stage of AD pathogenesis. These effects in turn interfere with neuronal function and result in progressive memory decline. A recent report using a goat anti-mouse VEGF polyclonal IgG also showed improved brain microvascular blood flow, reduced capillary stalling by neutrophils, and reduced BBB leakage in the cortex of APP/PS1 mice at 10–14 months of age, suggesting the therapeutic potential of targeting VEGF signaling for AD treatment [11]. It is worth noting the still controversial roles of VEGF and its impact on brain health in previous studies of AD mouse models. Strategies such as neuronal overexpression of VEGF, ventricle injection of bone marrow-derived mesenchymal stem cells expressing human VEGF, or direct supplementation of VEGF by brain injection of naked VEGF protein or encapsulated VEGF in biodegradable nanospheres have all been shown to improve memory performance and reduce amyloid plaques in APP/PS1 mice at 6–12 months of age [27, 103, 104]. In addition, intraperitoneal injection of VEGF protein has been shown to reduce neutrophil recruitment into APP/PS1 mice at 8–9 months of age [28]. These reports suggest the protective effects of ectopically delivered VEGF on the diseased brain of AD. Given the complex nature of VEGF function, it is suggested that fine tuning of VEGF signaling and its affecting cell types during the specific time window could play critical roles in determining the phenotypic outcomes of the disease.

Previous studies using VEGFR-targeting TKIs have suggested the pathological role of VEGF signaling in mouse models of AD. Male 5×FAD mice that received vatalanib treatment, a broad-spectrum TKI that targets VEGFR1, VEGFR2, platelet-derived growth factor receptor α (PDGFRα), and c-KIT, at 3–4 months of age showed reduced Aβ plaque burden and phosphorylated Tau levels in the cortex [33]. Similarly, axitinib, another TKI that also targets VEGF receptors, PDFR, and c-KIT, showed protective effects of reducing plaques and BBB leakage and improving memory performance in 10-month-old Tg2576 AD mice of both sexes [105]. These findings from TKI treatment have raised an important question of whether VEGF signaling is mainly responsible for the beneficial effects of TKIs or whether other VEGF-independent signaling pathways are also involved.

Furthermore, our study used the anti-VEGF antibody bevacizumab to specifically block VEGF function in 5×FAD mice at 4 months of age and demonstrated beneficial and significant effects of reducing amyloid-related brain pathology, improving vascular integrity and function, and rescuing memory performance. Bevacizumab was originally generated from a murine monoclonal antibody using a 165-amino-acid form of recombinant human VEGF as an immunogen [106]. The efficacy and biological effects of bevacizumab in inhibiting VEGF function and suppressing VEGF-mediated angiogenesis and vascular leakage have previously been reported in mouse models of diseases [36, 107–109]. Bevacizumab bound to both human and mouse VEGF proteins as shown by the surface plasmon resonance assay. However, a more than tenfold higher concentration of murine VEGF was necessary to reach a similar resonance unit as human VEGF for interaction with bevacizumab and the murine VEGF was prone to a more rapid dissociation than human VEGF [109]. Bevacizumab was found to inhibit angiogenesis in a mouse model of suture-induced corneal neovascularization, and was also effective but with lower efficacy than the anti-murine VEGF antibody in reducing late-onset tissue edema and necrosis in a mouse model of radiation-induced brain injury [36, 107–109]. However, the efficacy of bevacizumab in targeting mouse VEGF protein and its associated pathology has been questioned by a previous report [110], which could be due to the reasons of rapid dissociation kinetics of mouse VEGF protein from bevacizumab than human VEGF, the differences in mouse models examined, or the dosage and duration of drug administration.

Notably, peritoneally injected bevacizumab has been shown to enter the brain parenchyma and attenuate angiogenesis in mice after parenchymal infection with an adeno-associated viral vector overexpressing human VEGF [20]. Similar findings from studies showed that a therapeutically relevant concentration of human IgG can be detected in the cerebral tissues of mice with an intraperitoneal injection of a relatively high dose of human IgG (1.5 g/kg) into both a 15-month-old triple-transgenic AD mouse model (3 × Tg-AD) and nontransgenic littermates [111]. Whether an anti-VEGF monoclonal-based therapeutic strategy acts through blocking luminal VEGF or directly blocks parenchymal VEGF by crossing the BBB remains unknown and warrants further investigation. Since bevacizumab is a humanized monoclonal antibody and its safety and effectiveness in normalizing vasculature to facilitate cancer treatment in the last two decades have been clinically proven, our findings suggest the clinical potential of using bevacizumab in combination with other anti-Aβ monoclonal antibodies for the treatment of patients with mild cognitive impairment or at an early stage of AD [34].

As VEGFR1 and VEGFR2 are the main receptors that mediate VEGF function, increased cortical levels of VEGFR2 at 10–12 months of age in APP/PS1 and 5×FAD mice and decreased levels of VEGFR1 in the parietal cortex of AD patients and in the brains of 10- to 11-month-old male APP/PS1 mice have been reported previously [11, 23, 79]. However, whether changes in the protein levels of VEGFR1 and VEGFR2 in the brain parenchyma occur at the early stage of AD remains elusive. Our results unexpectedly revealed sex differences in VEGFR2 and sVEGFR1 protein levels in the brains of 5×FAD mice at 5 months of age. Specifically, we observed decreased levels of sVEGFR1 in both the cortex and hippocampus of female 5×FAD mice, whereas increased levels of VEGFR2 protein were found in both the cortex and hippocampus of male mice. Since sVEGFR1 protein has been considered a negative regulator of VEGF-mediated angiogenesis [23], our findings suggest that low protein levels of sVEGFR1 protein in female mouse brains may shift the balance to VEGFR2-dependent VEGF signaling, which is an effect comparable to the increased VEGFR2 protein levels found in the brains of male 5×FAD mice. Importantly, bevacizumab treatment restored the levels of sVEGFR1 and full-length VEGFR2 proteins in 5×FAD mice to a level similar to that in wild-type mice, suggesting that the loss of the balance and physiological strength of VEGF signaling in the brain parenchyma of AD patients and mouse models may be critical for the development of BBB damage and cerebrovascular dysfunction. It remains to be investigated which cell types expressing VEGFR1 and VEGFR2 proteins are involved in VEGF-mediated pathology and whether the manipulation of VEGF signaling in specific cell types may attenuate or accelerate the pathological process of AD.

By analyzing the transcriptomic profiles of the hippocampus of 5×FAD mice, the molecular signatures associated with bevacizumab treatment were investigated. By cross-referencing the RNA-seq data of the 5×FAD versus wild-type groups and the bevacizumab-treated 5×FAD versus vehicle-treated 5×FAD groups, we identified six shared DEGs with vascular-associated functions whose expression was altered in 5×FAD mice but reversed after bevacizumab treatment. Among them, *Cgn*, *Col3a1* and *Plagl1* were female-specific DEGs and *Pthlh*, *Efna5* and *Kcng3* were male-specific DEGs. *Cgn* is known to participate in the regulation of BBB integrity, and decreased *Cgn* expression has been shown to affect barrier function [81, 82]. Cytosolic cingulin in endothelial cells has been reported to form condensates with the tight junction protein ZO-1 that may facilitate the repair of junctional complexes [112]. Studies from cingulin knockout mice and knockdown experiments further showed that the loss of cingulin in endothelial cells resulted in morphologically intact tight junctions, but interestingly, studies also showed that cingulin knockout mice had increased endothelial leakage in the cerebellum [81]. In vitro studies showed that transiently knockdown of cingulin in endothelial cells caused an increase in endothelial permeability, while cingulin overexpression decreased endothelial permeability upon stimulation with histamine or VEGF-A. These findings suggest an important role of cingulin in maintaining endothelial barrier integrity under conditions of stress [113].

We also found that the protein levels of two endothelial tight junction proteins, ZO-1 and claudin 5, were unchanged in both sexes of 5×FAD mice compared with their wild-type littermates at 5 months of age. Reduced protein levels of ZO-1 have been reported in the brains of Tg2576 AD mice at 11 months of age in both sexes and can be restored by the pan VEGFR and tyrosine kinase inhibitor axitinib [105]. Reduced protein levels of claudin 5 were also reported in the cortex of male APP/PS1 mice at 8 months of age [28]. Our results therefore suggest that a decrease in the protein levels of cingulin may be involved in the early onset of hippocampal vascular leakage in female 5×FAD mice before the detectable dysregulation of other BBB-associated proteins, such as ZO-1 and claudin 5, which is a phenomenon that has not been reported previously.

Among the other identified DEGs, *Col3a1* encodes the alpha 1 chain of type III collagen, a major component of the extracellular matrix of blood vessels that is critical for vascular development and function [83, 84]. *Plagl1* is expressed in endothelial cells and acts as a transcription factor to regulate vasculature development [85]. *Efna5* encodes the ephrin-A5 protein, which binds to ephrin receptor A4 and plays a critical role in maintaining the density and diameter of microvessels in the adult hippocampus [88, 89]. Therefore, alterations in the expression of *Col3a1*, *Plagl1*, and *Efna5* could participate in abnormal vascular development, vessel density, and structural changes. *Pthlh* is expressed in endothelial cells and encodes a parathyroid hormone-related peptide that is antiangiogenic and exerts a vasodilatory effect in response to vasoconstriction [86, 87]. *Kcng3* is a potassium voltage-gated channel that has been suggested to play a role in the depolarization of vascular myocytes and hypertension [90, 91]. Downregulation of *Pthlh* and *Kcng3* may affect the vascular response and CBF. Alterations of these DEGs may therefore be involved in the early development of amyloidopathy-associated cerebrovascular pathology in 5×FAD mice.

Additional DEGs included the female-specific *Ifit1* and *Cox6b2*, which may be involved in the regulation of oxidative phosphorylation of mitochondria and interferon-induced cellular immune response [114–116]. Male-specific *C1qtnf1* is also associated with mitochondrial fission and collagen-induced platelet aggregation, which is known to protect against myocardial ischemic injury by inhibiting the inflammatory response through activation of the cyclic AMP signaling pathway [117–120]. Downregulation of *C1qtnf1* may therefore damage mitochondrial function and increase inflammation during the pathogenesis of AD.

GSEA further confirmed the enrichment of gene sets related to tight junction, focal adhesion, smooth muscle contraction, and Notch and Wnt signaling pathways that are functionally associated with the regulation of BBB integrity and vascular contraction [121–123]. Furthermore, the dysregulation of these pathways in the hippocampus of both female and male 5×FAD mice can be restored by bevacizumab treatment, which is in line with the improved vascular pathology in BBB leakage and cerebrovascular response to norepinephrine. Other gene sets that were differentially expressed in the 5×FAD mice and reversed by bevacizumab treatment were enriched in functions related to the regulation of mitochondrial function, electron transport, lipid metabolism, regulation of translation, and neural activity [124–129]. These findings are also in line with the therapeutic effects of bevacizumab that improved memory performance in 5×FAD mice.

## Conclusions

In summary, our study demonstrates that early intervention of VEGF function by bevacizumab effectively improved BBB integrity, the cerebrovascular response, and long-term memory in 5×FAD mice at 5 months of age. Sex-specific alterations in the protein levels of sVEGFR1 and full-length VEGFR2 in response to bevacizumab treatment were found in the cortex and hippocampus of 5×FAD mice. Analysis of the transcriptomic profiles further revealed bevacizumab-associated genes that are enriched for functional categories of vascular integrity and function. One major limitation of this study is the use of saline as a control treatment, which does not exclude the possibility of unspecific effects in addition to VEGF intervention that are contributed by the injected humanized antibody. Because bevacizumab has already been used in clinical treatment for patients with cancer and maculopathy, our findings suggest that bevacizumab may be considered a therapeutic option for early intervention of amyloidopathy-associated vascular pathology in patients with MCI or early-stage AD.

### Supplementary Information

**Additional file 1: Table S1.** Primer sequences used for RT-qPCR. **Fig. S1.** Effect of bevacizumab treatment on anxiety-like behavior of 5×FAD mice. **Fig. S2.** Bevacizumab treatment improves cerebrovascular responses to norepinephrine in both sexes of 5×FAD mice. **Fig. S3.** Unaltered density of cerebral blood vessels in bevacizumab-treated 5×FAD mice. **Fig. S4.** Blood pressure is unaffected in 5×FAD mice treated with bevacizumab. **Fig. S5.** Protein levels of VEGF in the hippocampus, cortex, and peripheral blood of 5×FAD mice. **Fig. S6.** Analysis of the transcriptomic profile reveals gene sets enriched in bevacizumab-treated 5×FAD mice.

## Data Availability

The datasets used and/or analyzed during the current study are available from the corresponding author on reasonable request. The sequencing data generated in this study have been deposited in the NCBI GEO database (tracking system #24087966).

## Abbreviations

AD: Alzheimer’s disease
Aβ: Amyloid beta
APP: Amyloid-beta precursor protein
PS1: Presenilin-1
VEGF: Vascular endothelial growth factor
VEGFR1: VEGF receptor 1
VEGFR2: VEGF receptor 2
sVEGFR1: Soluble VEGFR1
NOR: Novel object recognition
DI: Discrimination index
dHC: Dorsal hippocampus
S1: Primary sensory cortex
GFAP: Glial fibrillary acidic protein
IBA1: Ionized calcium-binding adapter molecule 1
CBF: Cerebral blood flow
RNA-seq: RNA-sequencing
*Cox6b2*: Cytochrome c oxidase subunit 6B2
*Col3a1*: Collagen, type III, alpha 1
*Ifit1*: Interferon-induced protein with tetratricopeptide repeats 1
*Plagl1*: Pleiomorphic adenoma gene-like 1
*Cgn*: Cingulin
*C1qtnf1*: C1q and tumor necrosis factor related protein 1
*Pthlh*: Parathyroid hormone-like peptide
*Efna5*: Ephrin A5
*Kcng3*: Potassium voltage-gated channel, subfamily G, member 3
ZO-1: Zonula occludens-1
