# Supplementary material for: Blockage of VEGF function by bevacizumab alleviates early-stage cerebrovascular dysfunction and improves cognitive function in a mouse model of Alzheimer’s disease
Source: Transl Neurodegener. 2024 Jan 3;13:1. doi: 10.1186/s40035-023-00388-4 (PMC10763201; doi:10.1186/s40035-023-00388-4)
Supplement: Supplementary file 1 — Additional file 1: Table S1. Primer sequences used for RT-qPCR. Fig. S1. Effect of bevacizumab treatment on anxiety-like behavior of 5×FAD mice. Fig. S2. Bevacizumab treatment improves cerebrovascular responses to norepinephrine in both sexes of 5×FAD mice. Fig. S3. Unaltered density of cerebral blood vessels in bevacizumab-treated 5×FAD mice. Fig. S4. Blood pressure is unaffected in 5×FAD mice treated with bevacizumab. Fig. S5. Protein levels of VEGF in the hippocampus, cortex, and peripheral blood of 5×FAD mice. Fig. S6. Analysis of the transcriptomic profile reveals gene sets enriched in bevacizumab-treated 5×FAD mice. [file 40035_2023_388_MOESM1_ESM.pdf]

Table S1 Primer sequences used for RT-qPCR

Gene	Forward primer	Reverse primer
Pthlh	CAACAAGGTGGAGACGTACAAA	CAGACCGAGTCCTTCGCTTC
Efna5	TCCAGAGGGGTGACTACCAC	GGCACTGTACCCATCAAAATTCA
Kcng3	GAACCCTCCGGGATAATTGAAG	CTCGCCTGTAAACACTGTCAT
C1qtnf1	TTTGCACAGCAACGACTACTT	CAGGTACGTCTCCTTCTGGTT
Plagl1	ATGGCTCCATTCCGCTGTC	CTCAGCCTTCGAGCACTTGAA
Cgn	CCAAGGCTAAGGTGGCATCA	CCTTCAACTGCTGCTCCAGA
Col3a1	ACGTAAGCACTGGTGGACAG	CAGGAGGGCCATAGCTGAAC
Ifit1	ATCGCGTAGACAAAGCTCTTC	GTTTCGGGATGTCCTCAGTTG
Cox6b2	CCAGAACCAGACGCGTAACT	AGGGTTGTGTGCTCTTTCCG
Gapdh	AGGTCGGTGTGAACGGATTTG	TGTAGACCATGTAGTTGAGGTCA

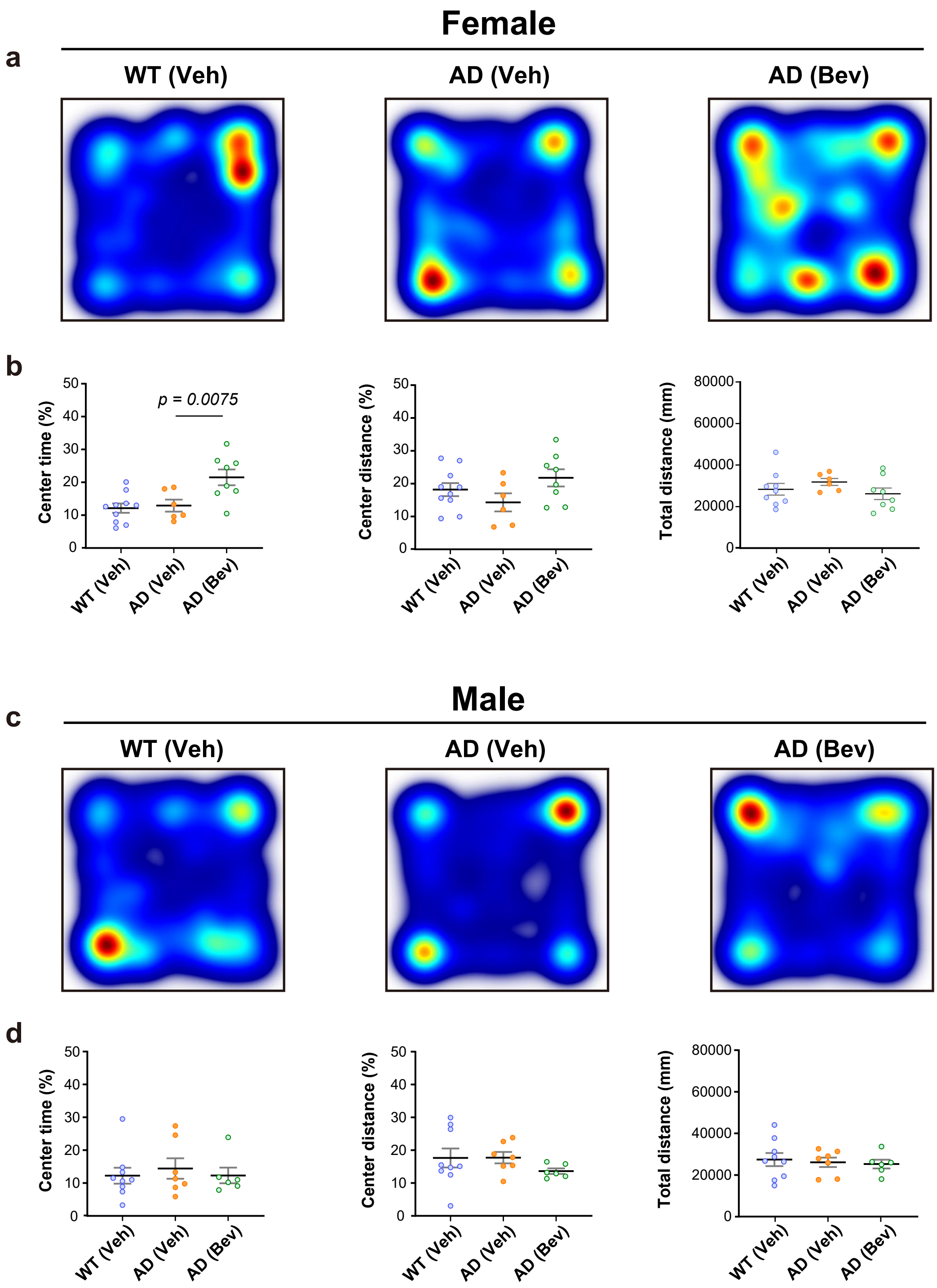

Supplementary Figure 1
Zhang et al

Supplementary Figure 1. Effect of bevacizumab treatment on anxiety-like behavior of 5×FAD mice

(a) Representative heatmaps of the traveling path of female mice in the open field test. (b) Quantitative analysis of the time spent in the center zone as a percentage of the total time, the distance traveled in the center zone as a percentage of the total distance traveled, and the total distance traveled in the open field across groups (n = 6-10 female mice per group). (c) Representative heatmaps of the traveling paths of male mice in the open field test. (d) Quantitative analysis of the time spent in the center zone as a percentage of the total time, the distance traveled in the center zone as a percentage of the total distance traveled, and the total distance traveled in the open field test (n = 6-9 male mice per group). All data are presented as the mean ± SEM and were analyzed by one-way ANOVA followed by Fisher's LSD test. WT (Veh): wild-type littermates receiving sham treatment, AD (Veh): 5×FAD mice receiving sham treatment, AD (Bev): 5×FAD mice receiving bevacizumab treatment.

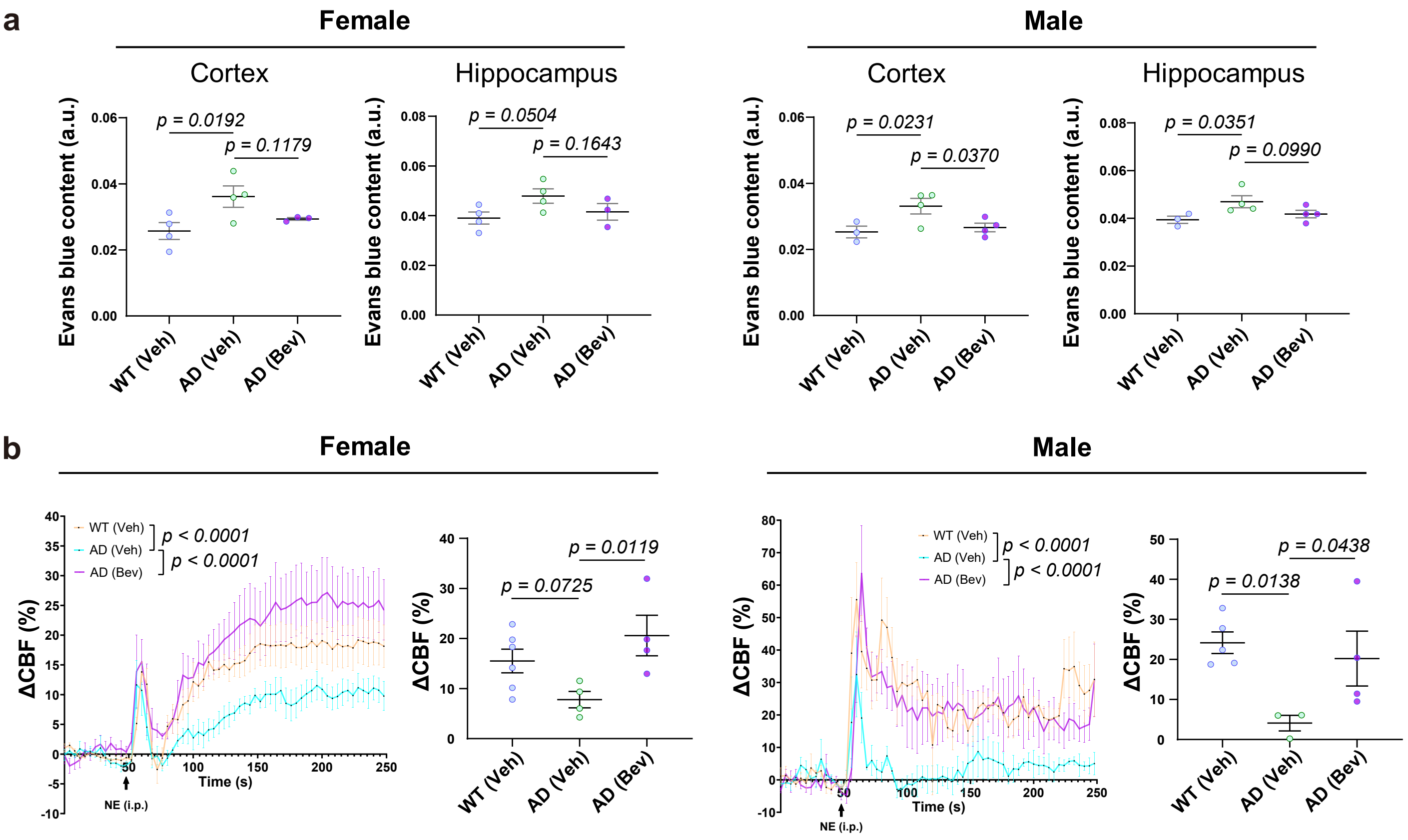

Supplementary Figure 2
Zhang et al

Supplementary Figure 2. Bevacizumab treatment improves cerebrovascular responses to norepinephrine in both sexes of 5×FAD mice

(a) Quantitative analysis of Evans blue dye leakage in the cortex and hippocampus of female and male 5×FAD mice. Evans blue content was normalized by the weight of the cortex or hippocampus, followed by normalization to the plasma concentration of Evans blue (n=3-4 mice per group). (b) Left panel for either sex: the curve graph showing the dynamic CBF changes (relative to the baseline CBF, △CBF) before and after norepinephrine injection. CBF changes in the WT (Veh), AD (Veh) and AD (Bev) groups at all time points were analyzed by two-way ANOVA followed by Tukey's multiple comparison test. Right panel for either sex: the sum of CBF changes from the 48 s time point (immediately before norepinephrine injection) to the 248 s time point was averaged to represent the CBF changes in each animal, and then the results were analyzed by one-way ANOVA followed by Fisher's LSD test. N=4-6 female mice per group, n=3-5 male mice per group. Data are presented as the mean±SEM. WT (Veh): wild-type littermates receiving sham treatment, AD (Veh): 5×FAD mice receiving sham treatment, AD (Bev): 5×FAD mice receiving bevacizumab treatment.

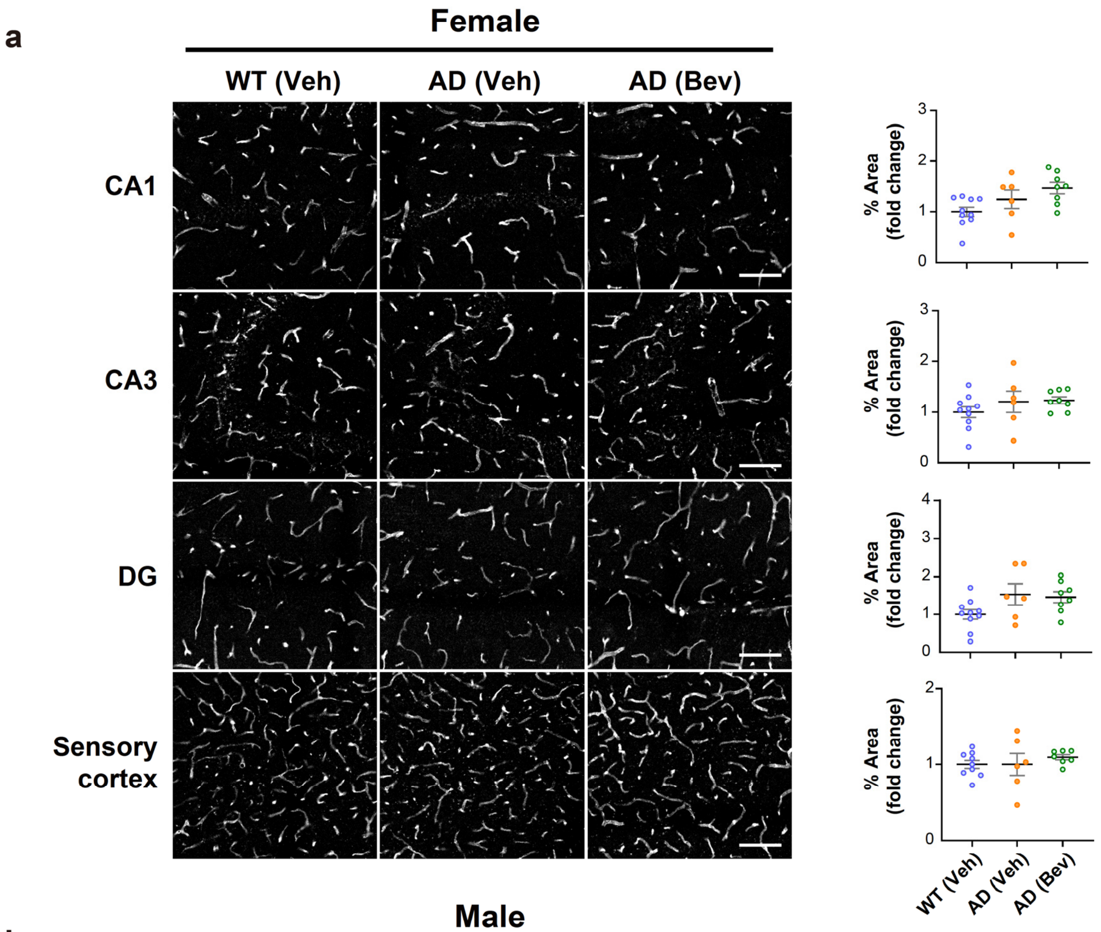

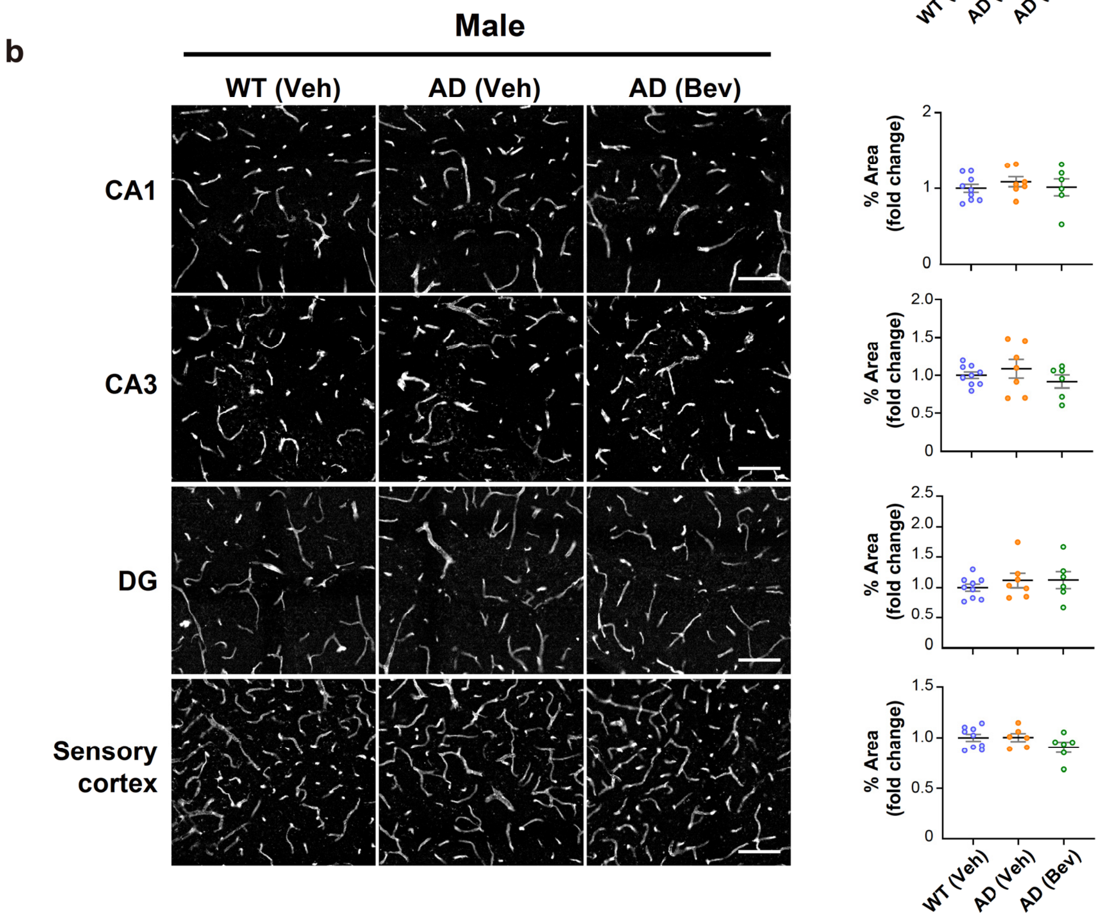

Supplementary Figure 3
Zhang et al

Supplementary Figure 3. Unaltered density of cerebral blood vessels in bevacizumab-treated 5×FAD mice

(a) Representative images of CD31 immunofluorescence staining, and quantitative analysis of CD31 fluorescence % area in the sensory cortex and the CA1, CA3 and DG subregions of the dorsal hippocampus of female mice (n=6-10 mice per group). Scale bar: 100 μm. (b) Representative images of CD31 immunofluorescence staining and quantitative analysis of CD31 fluorescence % area in the sensory cortex and the CA1, CA3 and DG subregions of the dorsal hippocampus of male mice (n=6-9 mice per group). Scale bar: 100 μm. All data are presented as the mean ± SEM and were analyzed by one-way ANOVA followed by Fisher's LSD test. WT (Veh): wild-type littermates receiving sham treatment, AD (Veh): 5×FAD mice receiving sham treatment, AD (Bev): 5×FAD mice receiving bevacizumab treatment CA1: cornu ammonis 1, CA3: cornu ammonis 3, DG: dentate gyrus.

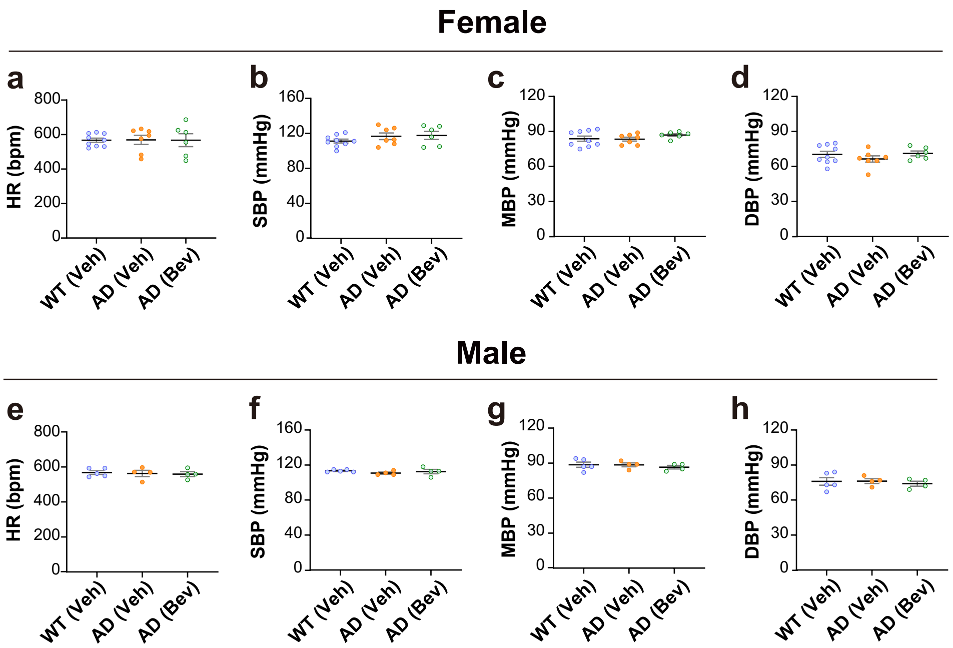

Supplementary Figure 4
Zhang et al

Supplementary Figure 4. Blood pressure is unaffected in 5×FAD mice treated with bevacizumab

(a-d) Quantitative analysis of (a) heart rate, (b) systolic blood pressure, (c) mean arterial blood pressure, and (d) diastolic blood pressure of female mice (n=6-9 mice per group). (e-h) Quantitative analysis of (e) heart rate, (f) systolic blood pressure, (g) mean arterial blood pressure, and (h) diastolic blood pressure of male mice (n=4-5 mice per group). All data are presented as the mean ± SEM and were analyzed by one-way ANOVA followed by Fisher's LSD test. WT (Veh): wild-type littermates receiving sham treatment, AD (Veh): 5×FAD mice receiving sham treatment, AD (Bev): 5×FAD mice receiving bevacizumab treatment. HR: heart rate, SBP: systolic blood pressure, MBP: mean arterial blood pressure, DBP: diastolic blood pressure, bpm: beat per minute, unit of heart rate, mmHg: unit of blood pressure.

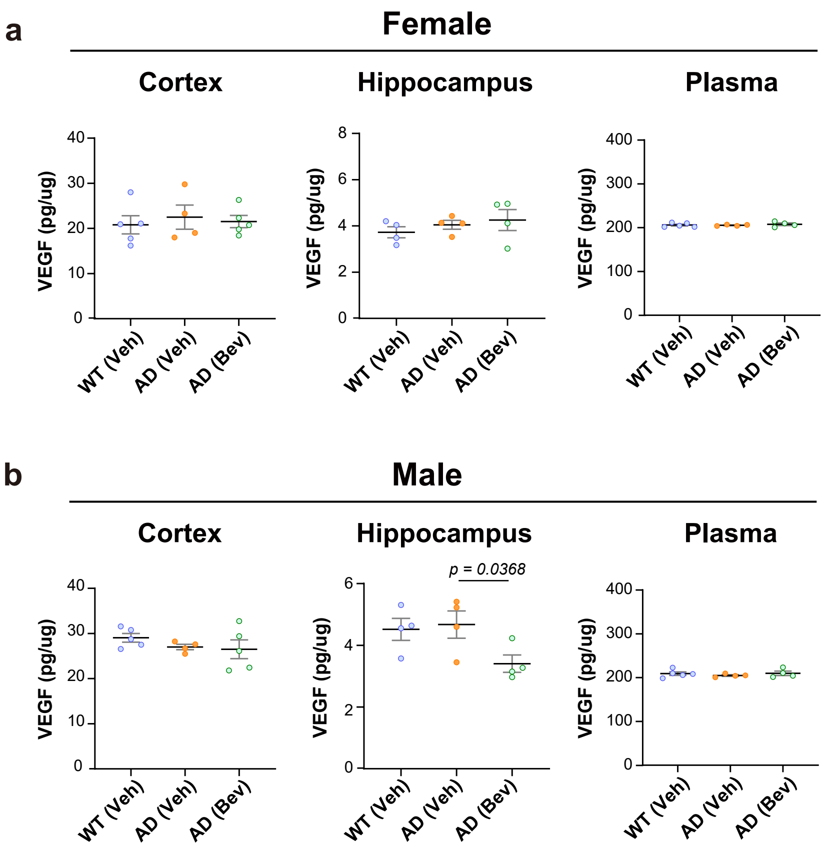

Supplementary Figure 5
Zhang et al

Supplementary Figure 5. Protein levels of VEGF in the hippocampus, cortex, and peripheral blood of 5×FAD mice

(a) VEGF protein levels in the cortex, hippocampus and plasma of female 5×FAD mice (n=4-5 mice per group). (b) VEGF protein levels in the cortex, hippocampus and plasma of male 5×FAD mice (n=4-5 mice per group). All data are presented as the mean ± SEM and were analyzed by one-way ANOVA followed by Fisher's LSD test. WT (Veh): wild-type littermates receiving sham treatment, AD (Veh): 5×FAD mice receiving sham treatment, AD (Bev): 5×FAD mice receiving bevacizumab treatment.

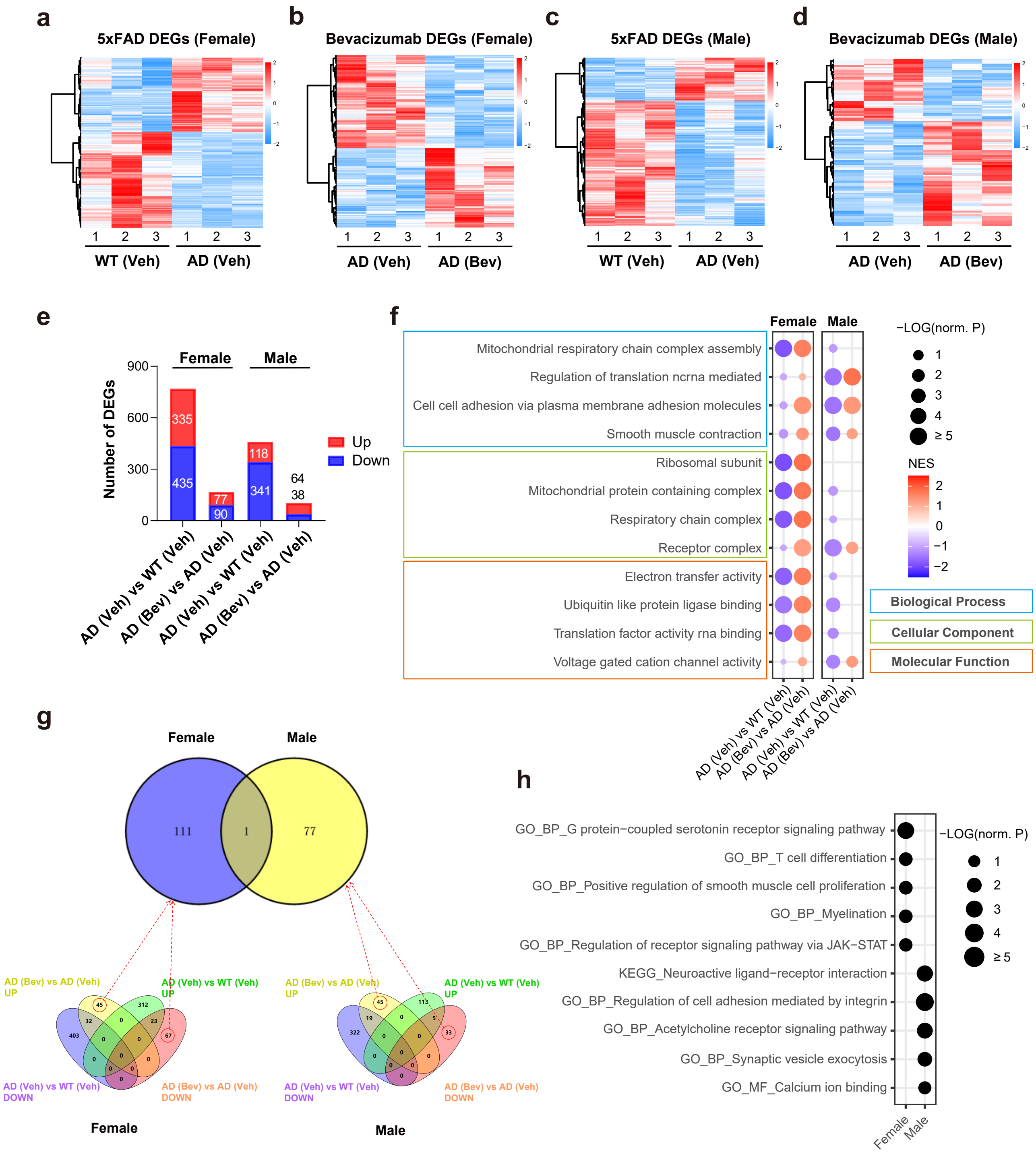

Supplementary Figure 6

Zhang et al

Supplementary Figure 6. Analysis of the transcriptomic profile reveals gene sets enriched in bevacizumab-treated 5×FAD mice

(a-d) Heatmaps showing DEGs in the hippocampus of 5×FAD mice. (a) DEGs of the female wild-type vehicle-treated group vs. the female AD vehicle-treated group. (b) Female AD vehicle-treated group vs. female AD bevacizumab-treated group. (c) Male wild-type vehicle-treated group vs. male AD vehicle-treated group. (d) Male AD vehicle-treated group vs. male AD bevacizumab-treated group. (e) The summarized DEG number from the comparison of different groups. (f) GO pathway analysis based on GSEA was performed for RNA-seq data of the female and male 5×FAD mice. (g) The transcriptomic profiles of bevacizumab-specific DEGs. Venn diagram depicting limited overlaps of the DEGs of the female and male groups. (h) Pathways analysis of KEGG and GO based on GSEA was performed for RNA-seq data of the female and male 5×FAD mice. WT (Veh): wild-type littermates receiving sham treatment, AD (Veh): 5×FAD mice receiving sham treatment, AD (Bev): 5×FAD mice receiving bevacizumab treatment.
